# Implications of Anion Structure on Physicochemical Properties of DBU-Based Protic Ionic Liquids

**DOI:** 10.1021/acs.jpcb.2c02789

**Published:** 2022-08-30

**Authors:** Giselle de Araujo Lima e Souza, Maria Enrica Di Pietro, Franca Castiglione, Pedro Henrique Marques Mezencio, Patricia Fazzio Martins Martinez, Alessandro Mariani, Hanno Maria Schütz, Stefano Passerini, Maleen Middendorf, Monika Schönhoff, Alessandro Triolo, Giovanni Battista Appetecchi, Andrea Mele

**Affiliations:** † Department of Chemistry, Materials and Chemical Engineering “Giulio Natta”, Politecnico di Milano, Piazza Leonardo da Vinci 32, 20133 Milan, Italy; ‡ School of Chemical Engineering, University of Campinas, Street Albert Einstein 500, 13083-852 Campinas, Brazil; § Università Politecnica Delle Marche, Piazza Roma, 22, 60121 Ancona, Italy; ∥ Helmholtz Institute Ulm (HIU), Helmholtzstraße 11, D-89081 Ulm, Germany; ⊥ Karlsruhe Institute of Technology (KIT), P.O. Box 3640, D-76021 Karlsruhe, Germany; # Institute of Physical Chemistry, University of Muenster, Corrensstrasse 28-30, 48149 Münster, Germany; % Istituto Struttura della Materia (ISM), Consiglio Nazionale delle Ricerche (CNR), via Fosso del Cavaliere 100, 00133 Rome, Italy; & ENEA (Italian National Agency for New Technologies, Energy and Sustainable Economic Development), Department for Sustainability (SSPT), Casaccia Research Center, Via Anguillarese 301, 00123 Rome, Italy

## Abstract

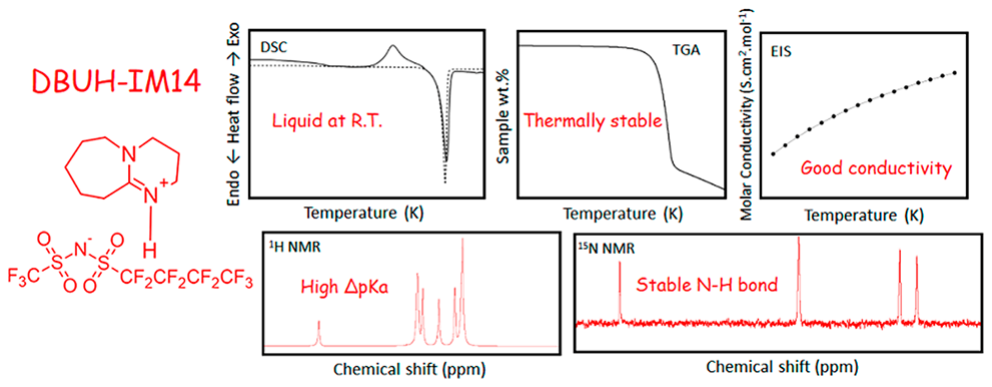

Protic ionic liquids (PILs) are potential candidates as electrolyte components in energy storage devices. When replacing flammable and volatile organic solvents, PILs are expected to improve the safety and performance of electrochemical devices. Considering their technical application, a challenging task is the understanding of the key factors governing their intermolecular interactions and physicochemical properties. The present work intends to investigate the effects of the structural features on the properties of a promising PIL based on the 1,8-diazabicyclo[5.4.0]undec-7-ene (DBUH^+^) cation and the (trifluoromethanesulfonyl)(nonafluorobutanesulfonyl)imide (IM14^–^) anion, the latter being a remarkably large anion with an uneven distribution of the C–F pool between the two sides of the sulfonylimide moieties. For comparison purposes, the experimental investigations were extended to PILs composed of the same DBU-based cation and the trifluoromethanesulfonate (TFO^–^) or bis(trifluoromethanesulfonyl)imide (TFSI^–^) anion. The combined use of multiple NMR methods, thermal analyses, density, viscosity, and conductivity measurements provides a deep characterization of the PILs, unveiling peculiar behaviors in DBUH-IM14, which cannot be predicted solely on the basis of differences between aqueous p*K*_a_ values of the protonated base and the acid (Δp*K*_a_). Interestingly, the thermal and electrochemical properties of DBUH-IM14 turn out to be markedly governed by the size and asymmetric nature of the anion. This observation highlights that the structural features of the precursors are an important tool to tailor the PIL’s properties according to the specific application.

## 1 Introduction

Ionic liquids (ILs) are potential candidates to replace organic solvents in electrochemical applications due to their interesting characteristics such as low flammability, low vapor pressure, high ionic conductivity, and high thermal stability.^1^ Within the ever-growing family of ILs, the role of protic ionic liquids (PILs) is gaining popularity in the field of energy storage devices since the past decade.^2^ According to Greaves and Drummond,^3^ PILs are a subset of ILs that are prepared through the stoichiometric neutralization reaction of certain Brønsted acids and Brønsted bases, having an available proton on the cation.

To promote the practical industrial application of PILs as electrolytes in energy storage devices,^4^ an all-embracing understanding of the system is needed to correlate with, explain, and ideally predict properties and performance of the materials. First, the presence of an acidic proton in the bulk liquid introduces different ion–ion interactions along with the conventional Coulombic and dispersion forces.^5^ Hence, investigations at the molecular level are needed to provide information about the interspecies interactions, leading to hints about their molecular structure and organization. Macroscopic properties also need to be well-defined, including density, viscosity, thermal, and electrochemical behavior. Indeed, PILs emerge as promising alternative to conventional organic solvents used as electrolytes, being endowed with high safety, thermal and electrochemical stability, and appreciable conductivity up to 30 mS cm^–1^.^6^ However, the aspects governing their macroscopic and transport properties are not well understood up to now. In this framework, the ionicity has been proposed to access the effective fraction of ions participating in the conduction process.^7^ According to the Walden rule,^8^ the liquid viscosity offers frictional resistance to charge mobility, and consequently, the molar conductivity of a solution is proportional to its fluidity. The ionicity of a PIL can be estimated through its deviation from the ideal behavior of a fully dissociated electrolyte.^9^ Reduced ionicity values are often related to the presence of ion-pairing and/or aggregates.^9,10^ Other factors such as interacting ions present in the liquid structure,^11^ charge transfer between cation and anion,^12^ and anticorrelation motions can also play a crucial role in the conductivity of PILs.^13^ Another important parameter claimed to govern the properties of a PIL is its Δp*K*_a_,^14^ which is defined as the difference between the aqueous p*K*_a_ values of the protonated base and the acid.^8^ The Δp*K*_a_ is commonly related to the driving force for the proton transfer from a Brønsted acid to a Brønsted base during the synthesis of a PIL. For systems based on the superstrong base 1,8-diazabicyclo[5.4.0]undec-7-ene (DBU), usually Δp*K*_a_ values equal to or higher than 15 give stable PILs,^14^ but the threshold value is still under discussion.^15^ Although a large part of the scientific community still consider high Δp*K*_a_ values as an indicator of thermal stability and high ionicity in PILs,^14^ it has been demonstrated that structural features also play a crucial role in governing the properties of a PIL.^15^ Moreover, the p*K*_a_ values are meaningful only in aqueous systems, and the approach does not take into account the acid strength in the IL environment.^16^ This is why proton affinity (PA) has been suggested as a more reliable indicator of the degree of proton transfer,^17^ with lower PA of the anion translating into stronger associated acid, but it is unclear how it correlates with the macroscopic behavior of the whole system yet.

**Figure 1:**
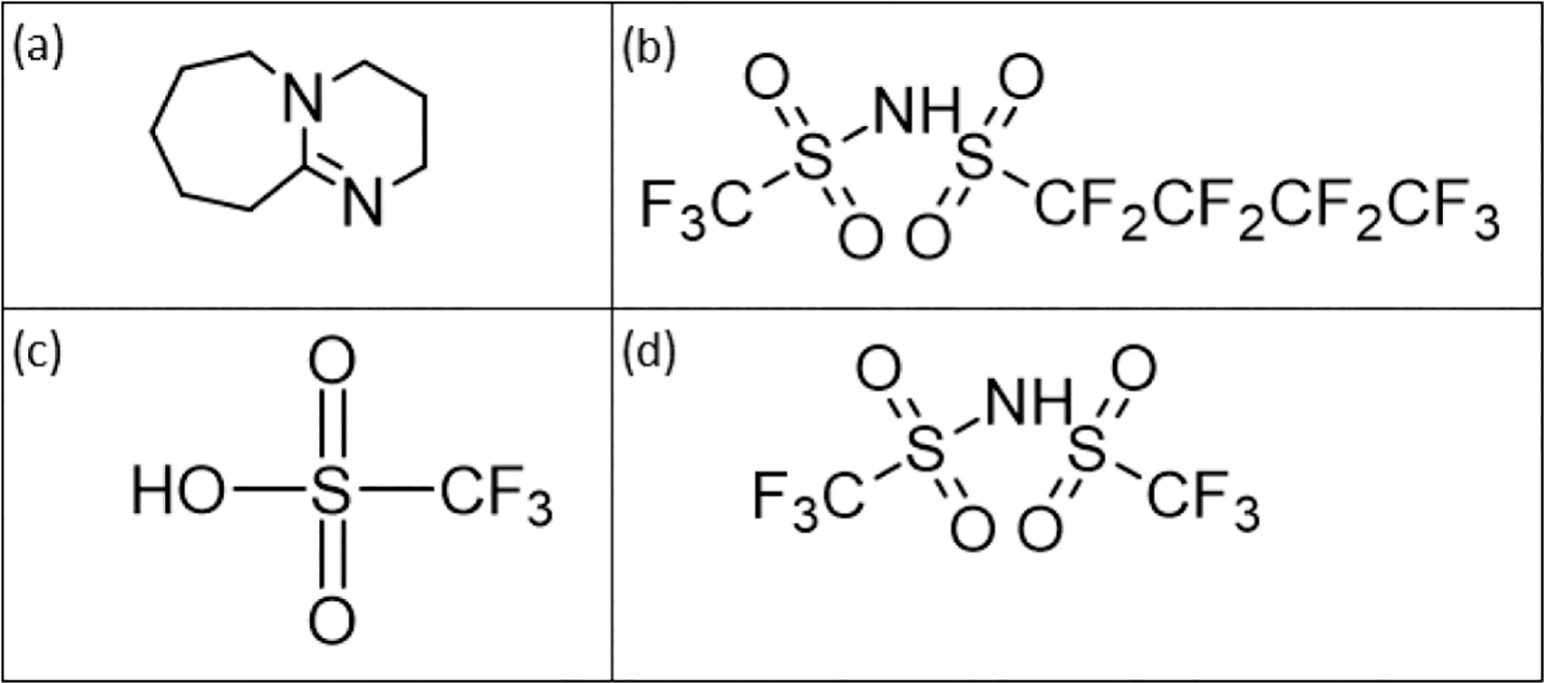
Structure of the PILs’ precursors: DBU base (a) and the three acids HIM14 (b), HTFO (c), and HTFSI (d).

In this evolving scenario, it is evident that the anion strongly affects the transport properties of a PIL and a complex interplay between size and shape of the anion on the one side and degree of proton transfer from the other must be considered for a comprehensive understanding of the PILs’ features. To contribute to disentangling this interplay, we selected the 1,8-diazabicyclo[5.4.0]undec-7-ene (DBU) base and the (trifluoromethylsulfonylnonafluorobutylsulfonyl)imide acid (HIM14) as precursors for PIL synthesis. DBU is a superbase (p*K*_a_ = 13.4)^14^ characterized by having a large size and strong charge delocalization on the N–C=N moiety.^18^ HIM14 is a markedly large anion having an uneven distribution of the C–F pool between the two sides of the sulfonylimide moieties, with nonavailable p*K*_a_ and PA but expected acidic strength comparable to other similar imide superacids (expected p*K*_a_ ∼ −10)^19^ (Figure 1). The properties of DBUH-IM14 have been compared to the DBU-based PILs formed by two other anions of very strong acids: trifluoromethylsulfonic (HTFO, p*K*_a_ = −7, PA = 305 kcal/mol)^14,17^ and bis(trifluoromethylsulfonyl)imide (HTFSI, p*K*_a_ = −10, PA = 294 kcal/mol).^14,17^ Selecting PILs with similar (and very high) acidity but different anion structure ensures the complete proton transfer in all three systems and is crucial to disentangle the influence of the structure of the anion and the acidity of the precursors in the properties of the PIL. Indeed, the appealing thermal properties, ionic conductivity, and ionicity of DBUH-IM14 differ from the results expected solely on the base of the acidity. This work evidences that geometrical features, H-bond acceptor capability, steric hindrance, and tendency to form fluorophilic domains of the IM14^–^ anion^19,20^ play a crucial role in dictating the highly appealing physicochemical characteristics of the corresponding PIL.

## 2 Methods

Three PILs—DBUH-IM14, DBUH-TFSI, and DBUH-TFO—were prepared following an established protocol,^21,22^ consisting of a standard neutralization reaction (for DBUH-IM14 and DBUH-TFO) or a neutralization followed by a metathesis reaction (for DBUH-TFSI). Additional purification and vacuum-drying steps yielded dry pure systems with 1:1 stoichiometry. All samples were characterized by means of ^1^H and ^15^N NMR spectroscopy, differential scanning calorimetry (DSC), thermogravimetric analysis (TGA), density and viscosity measurements, and electrochemical impedance spectroscopy (EIS). Details on sample preparation and purification as well as characterization methods are given in the Supporting Information.

## 3 Results and Discussion

### 3.1 ^1^H and ^15^N NMR Measurements

The Introduction highlighted the key role of PIL constituents as a factor governing their physicochemical properties.^14^ The acidity of the very strong acid HIM14 is expected to be comparable to HTFSI,^19^ but the exact value is still missing in the literature. To fill this gap, we indirectly quantified this acidity of the HIM14 via ^1^H NMR and ^15^N NMR , which afford structural information about the protonation of the base that is in turn strictly related to the acid strength.

**Figure 2:**
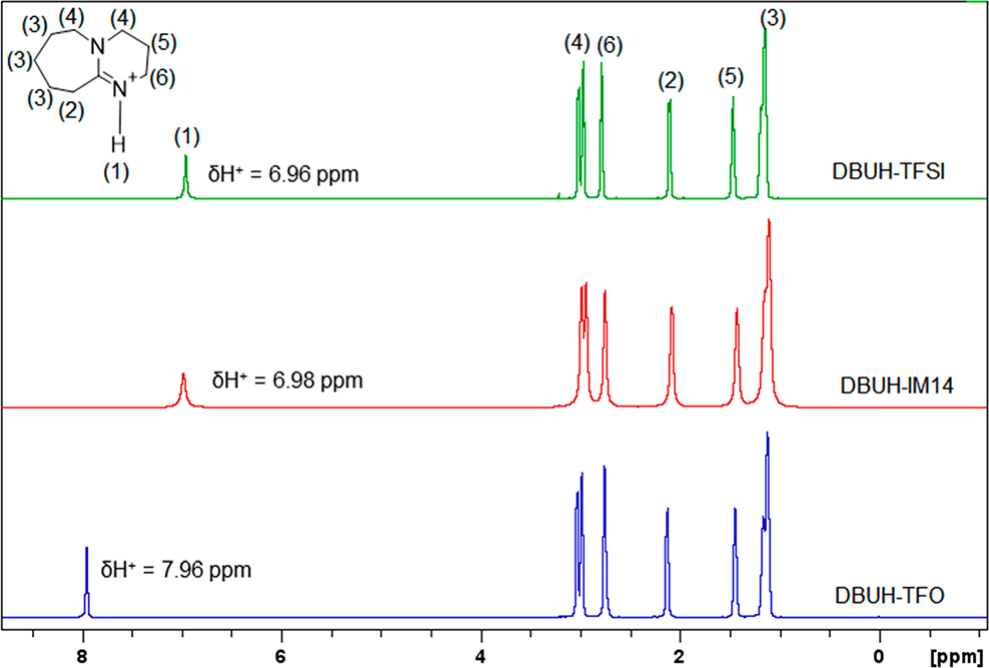
^1^H NMR spectra and chemical shift assignment of the PILs investigated at 328 K.

According to the literature,^14,17^ the ^1^H chemical shift changes linearly with the acid strength. Figure 2 displays the ^1^H NMR spectra and the chemical shift assignment of the PILs investigated at 328 K. The ^1^H signals appearing in the spectral region between 1 and 3 ppm are assigned to the C–H protons of the DBUH backbone (molecular formula and atom numbering in Figure 2). The most deshielded signal corresponds to the acidic N–H proton available on the DBUH^+^ component. As shown in Figure 2, the chemical shift of the exchangeable proton is decreased (shielding effect; i.e., it moves upfield or to lower frequencies) as the Δp*K*_a_ of the PIL increases and the PA of the acid decreases (see Table 1). The upfield shift experienced by the N–H proton is related to the extent of the acidic dissociation of the exchangeable proton attached to the imine nitrogen. Accordingly, the chemical shift difference between the acidic proton and the most shielded proton, i.e., the CH_2_ (3) group of the DBU,^23^ has been chosen as a marker of protonation and an indicator of the acid strength (see Table 1). Remarkably, no signal was detected in the 10–20 ppm spectral range, ruling out the presence of the free acid in the system^24^ (Figure S1).

**Table 1:** Correlation between ^1^H and ^15^N NMR Resonances with Δp*K*_a_ of the PILs Studied or PA of the Corresponding Anion^a^

PIL	Δδ(^1^H)^b^ (ppm)	Δδ(N2)^c^ (ppm)	Δp*K*_a_	anion PA (kcal/mol)
DBUH-TFO	6.81	100.6	20.4^d^	305^e^
DBUH-TFSI	5.80	101.8	23.4^d^	294^e^
DBUH-IM14	5.85	101.8	≃23.4	≃294

a Standard uncertainties *u* are *u*(Δδ(^1^H)) = 0.05 ppm; *u*(Δδ(N2)) = 0.1 ppm and *u*(*T*) = 0.1 K.

b Δδ(^1^H) = δH^*+*^– δCH_2_(3) at 328 K.

c Δδ(N2) = |δ(N2)_(cation)_ – δ(N2)_(free-base)_| at *T* = 308 K.

d Reference (14).

e Reference (17).

**Figure 3:**
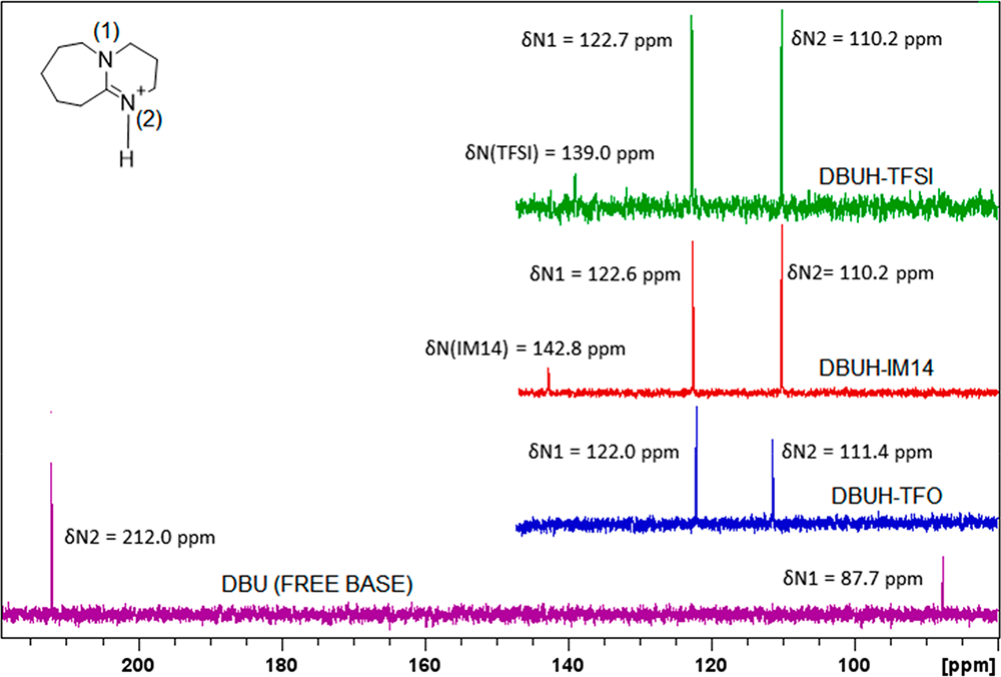
1D ^15^N–{^1^H} NMR spectra and the chemical shift assignments of the PILs investigated at 308 K.

The proton-decoupled ^15^N NMR spectra recorded at 308 K are displayed in Figure 3. The spectrum of the free base (DBU) shows two signals at 87.7 and 212.0 ppm assigned to the amino (N1) and imino (N2) nitrogen, respectively.^24^ Upon protonation, the signal of the imino (N2) nitrogen is significantly shielded (110.2, 110.2, and 111.4 ppm for DBUH-IM14, DBUH-TFSI, and DBUH-TFO, respectively), while the amino nitrogen (N1) in the β position with respect to the protonation site shows a strong downfield shift (122.6, 122.7, and 122.0 ppm for DBUH-IM14, DBUH-TFSI, and DBUH-TFO, respectively), in agreement with the charge and electron density delocalization onto the N–C=N moiety. A similar trend was shown by Angell et al. in the case of the protonation of 1,3-dimethyl-2-imidazolidinone (DMI).^25^ The ^15^N chemical shift variation at N2 between the protonated and the free base is around 100 ppm (see Table 1), confirming the full protonation of the imino nitrogen.^24^ The nitrogen nuclei belonging to IM14^–^ and TFSI^–^ were detected at 142.8 and 139.0 ppm, respectively.

The observed trends in ^1^H and ^15^N chemical shift data are in line with the known and expected proton donor ability of the acids and proton acceptor character of the DBU base, as shown in Table 1. Indeed, the chemical shift differences Δδ(^1^H) and Δδ(^15^N at N2) of DBUH-IM14 are very close to those of DBUH-TFSI, indicating that the HIM14 acidity is comparable to that of HTFSI. This finding is particularly relevant as p*K*_a_ and PA of HIM14 have not been reported yet and considering that HIM14 is an important precursor of PILs with high thermal stability. Also, it is worth pointing out that DBUH-IM14 is liquid at room temperature, likely due to the asymmetry of the fluorinated chains linked to the sulfonamide group in the IM14^–^ anion. Other DBU-based PILs having very strong acids as a precursor, such as DBUH-TFSI and DBUH-TFO, are actually solid at room temperature (see Table 2), thus hampering their technical application.^19^

**Table 2:** Thermal Characterization of the Studied PILs

PIL	*T*_g_ (K)	*T*_c-c_ (K)	*T*_m_ (K)	*T*_d_^a^ (K)	*T*_d_^b^ (K)
DBUH-TFO	197.9	269.5	303.6	759.6	753.3
	212.1^c^		296.2^c^	704.5^c^	
DBUH-TFSI			301.1	762.3	758.3
			298.2^c^	724.2^c^	
DBUH-IM14	201.4	247.1	290.9	712.4	713.3

a Under a nitrogen atmosphere.

b Under a synthetic air atmosphere.

c Data from ref (14).

The 1D ^15^N NMR spectra run without proton decoupling shown in Figure S3 serve as unambiguous evidence for the exclusive protonation of the imino nitrogen. In all samples, the signal appearing around 110 ppm is a well-resolved doublet with a one-bond ^1^*J*_N–H_ scalar coupling constant of about 98 Hz, which is consistent with the formation of the N–H covalent bond upon protonation.^24^ Thus, the doublet multiplicity of the N2 signal is the direct spectroscopic evidence of the formation of the stable DBUH^+^ cation on the NMR time scale investigated for all the PILs studied. From a kinetic viewpoint, the detection of stable and well-defined signal splitting due to ^1^*J*_N–H_ scalar coupling means that the rate of proton exchange is slower than the time scale associated to the spin–spin coupling, i.e., here slower than ∼10 ms. This implies that the N–H moiety is also kinetically stable and fully available as a H-bond donor.

This kinetic stability is also maintained in the ^15^N–{^1^H} NMR spectra acquired as a function of increasing temperature. The ^15^N chemical shift of the amino and imino nitrogen measured as a function of temperature is shown in Figure S4. Only a small linear variation in the ^15^N chemical shift is detectable in both signals assigned to nitrogen nuclei of DBUH^+^ (Figure S4a,b). No significant or discontinuous ^15^N chemical shift change of the nitrogen signal of TFSI^–^ and IM14^–^ anions was detected in the same temperature range (Figure S4c). The main conclusion is that the extent of protonation remains constant with increasing temperature, indicating that imino nitrogen of DBU base (N2) is fully protonated within the explored temperature range.

**Figure 4:**
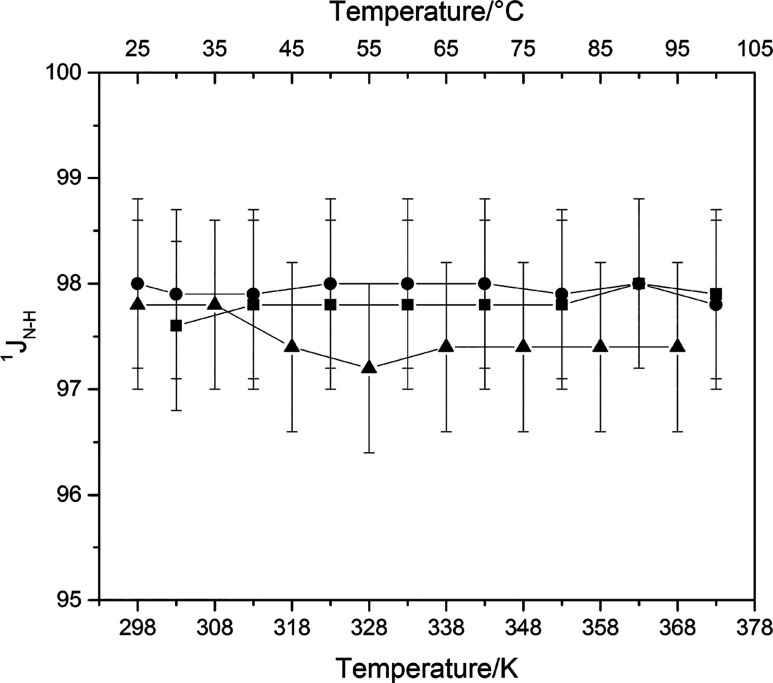
^1^*J*_N–H_ (Hz) of the imino nitrogen for the (■) DBUH-TFSI, (●) DBUH-IM14, and (▲) DBUH-TFO as a function of the temperature. Standard uncertainties *u* are *u*(^1^*J*_N–H_) = 0.8 Hz and *u*(*T*) = 0.1 K.

The thermal stability of the N–H bond between the imino nitrogen and the acidic proton is also confirmed by INEPT experiments (Figure 4 and Figures S5–S7), which revealed an approximately constant ^1^*J*_N–H_ in the temperature range investigated for all the PILs studied. Thus, the INEPT evidenced the thermal stability of the N–H bond in the full temperature range evaluated, confirming that the proton residence time on the imino N remained above the order of the 10 ms range in the explored temperature range. The extensive NMR evaluation performed provides evidence that DBUH-IM14 is formed by a very strong acid, favoring the complete proton transfer and leading to a system with a negligible content of neutral species and stable in the whole temperature interval investigated.

### 3.2 Thermal Analyses

Once the formation of a fully ionic system is assessed, we investigated how the structural features of the IM14^–^ anion affect physical chemical properties of this PIL. The thermal characterization of PILs is relevant because their physical state and thermal stability might limit the target application. In the present work, the thermal events of DBU-IM14 were evaluated for the first time through DSC (Figure S8) and TGA (under N_2_ atmosphere (Figure S8) and under synthetic air (Figure S9)) measurements and compared with the thermal features of the PILs DBUH-TFSI and DBUH-TFO, as summarized in Table 2.

The first heating scan of DBUH-IM14 shows a glass transition (*T*_g_) at 201.4 K, followed by the cold crystallization (*T*_c-c_) at 247.1 K and finally the melting transition at 289.8 K (Figure S8a, solid line). In the cold crystallization, a subcooled liquid crystallizes upon heating above the glass transition state to a crystalline solid; such a kind of thermal event has already been reported in the case of some TFSI containing ILs, for example.^26^ The cold crystallization event, ascribed to a not full crystallization of the sample, can be associated with intermolecular interactions and to the possibility of a multiplicity of readily accessible conformational rearrangements.^27^ For the DBUH-IM14, the cold crystallization feature observed at 10 K min^–1^ can be related to its asymmetrical fluorine-containing chain organization (CF_3_ and C_4_F_9_ moieties are located on the opposite sides of the dialkylsulfonylimide frame), its conformational flexibility, and the capability of IM14^–^ anions to generate fluorophilic domains, as previously highlighted.^19,20,28−33^ It is worth highlighting that IM14-based ILs generally exhibit no solid-phase transition (i.e., no melting feature) as the high asymmetry of the IM14^–^ anion prevents the crystallization process.^34^ However, in the case of the DBUH-IM14 a fully crystalline system was obtained by applying several refrigeration cycles. At those conditions (Figure S8a, dotted line), the melting point of DBUH-IM14 slightly changed to 290.9 K.

At odds with DBUH-IM14, DBUH-TFSI (Figure S8b) cannot be supercooled into its glassy state, at least at the presently chosen cooling rates. This behavior is quite common for TFSI-based ILs and can be related to the symmetric nature of the anion geometry that easily drives the sample toward its crystalline phase, although, given the distributed nature of electronic charge, rather low melting points are generally observed.^35^ Here, the thermal profile of DBUH-TFSI (Figure S7b) shows the melting at 301.1 K, in good agreement with the value 298.2 K reported by Miran et al.,^14^ considering the different scan rate used in both cases.

The DSC curves recorded for the DBUH-TFO (Figure S8c) indicate that the thermal history substantial affects its thermal properties. The glass transition (*T*_g_ = 197.9 K) observed during the first heating scan (Figure S8c, solid line) suggests that this sample tends to crystallize only at a slow cooling rate, thus allowing the formation of a glassy state, when cooling at high enough rate. The slightly different value of *T*_g_ (212.1 K) reported in the literature^14^ compared to the one determined here can be explained by the different thermal histories during the crystallization of the sample. Upon heating, above the glass transition, the supercooled liquid undergoes a cold crystallization (*T*_c-c_ = 269.5 K) and finally the melts at 297.6 K, which well matches the value of 296.2 K reported by Miran et al.^14^ To precisely determine the melting point of DBUH-TFO, refrigeration cycles were performed to fully crystallize the sample. The fully crystalline state was achieved once the glass transition and the cold crystallization features disappeared in the last heating scan (Figure S8c, dashed line). The DSC trace reveals a solid–solid phase transition at 253.5 K, already reported in the case of triflate-based ILs.^36^ Finally, the sample undergoes melting at 303.6 K. The increase of 7.7 K in the melting point when the sample is fully crystallized is related to the reduction of the mobility of the ions due to the confined effect produced by the crystallization and the highly oriented hydrogen bonds.^37,19^

The different melting temperatures of the three PILs indicate the influence of the ion sizes and the intermolecular interactions on the phase transition temperature. In the fully crystalline state, the melting temperature follows the order DBUH-TFO > DBUH-TFSI > DBUH-IM14. Considering the nature of the intermolecular interactions on these PILs, one would expect a similar melting point for the DBUH-TFSI and DBUH-IM14. However, a crucial aspect is introduced here by the large and asymmetrical IM14^–^ anion because it hinders lattice formation, consequently decreasing the melting temperature of the system.^38^ From a practical point of view, DBUH-IM14 has an advantage with respect to the other systems because it is liquid at room temperature. This benefit expands its possible range of applications, such as nonvolatile electrolytes components,^39^ as a solvent for reactions^40^ and separations processes,^41^ and heat transfer fluids.^42^

For the above-mentioned prospective applications, DBUH-IM14 is also expected to have good thermal stability. Figures S8 and S9 display the dynamic TGA traces of the three PILs measured in nitrogen and synthetic air atmospheres, respectively. The decomposition temperatures (*T*_d_) were obtained from the DTG curve reported in the Supporting Information (Figures S10 and S11). In both circumstances, the decomposition process occurs around 710 K for DBUH-IM14 and 760 K for DBUH-TFSI and DBUH-TFO. Then, the thermal stability of the selected PILs follows the order DBUH-TFSI ≈ DBUH-TFO > DBUH-IM14. The difference between the results reported here and the literature^14^ might be due to the different impurities content of the samples which have a catalytic effect on the decomposition process.^43^

To further investigate the thermal behavior of the PILs under a prolonged heating time, isothermal TGA experiments were performed in synthetic air (Figure S12). The results reveal lower stability of the PILs with respect to that reported by the dynamic TGA experiments. For instance, a practical weight loss is observed already above 523, 546, and 573 K for DBUH-IM14, DBUH-TFO, and DBUH-TFSI, respectively.

In all the scenarios of investigation, the DBUH-IM14 was the least thermally stable system, while, with DBUH-IM14 being characterized by a degree of proton transfer as high as DBUH-TFSI (*vide supra*), one might have expected comparable temperature of degradation of these two systems. Indeed, a correlation between degradation temperature (*T*_d_) values and the Δp*K*_a_ of a PILs is reported in the literature.^14^ However, for the DBUH-IM14, the perfluoroalkyl chains seem to play a crucial role in decreasing the lattice potential energy and consequently lowering the thermal stability of this PIL. For instance, a lower thermal stability of IM14-based ionic liquids, as compared to the analogous TFSI ones (sharing the same cation), was previously observed also in ammonium ILs.^44^

**Figure 5:**
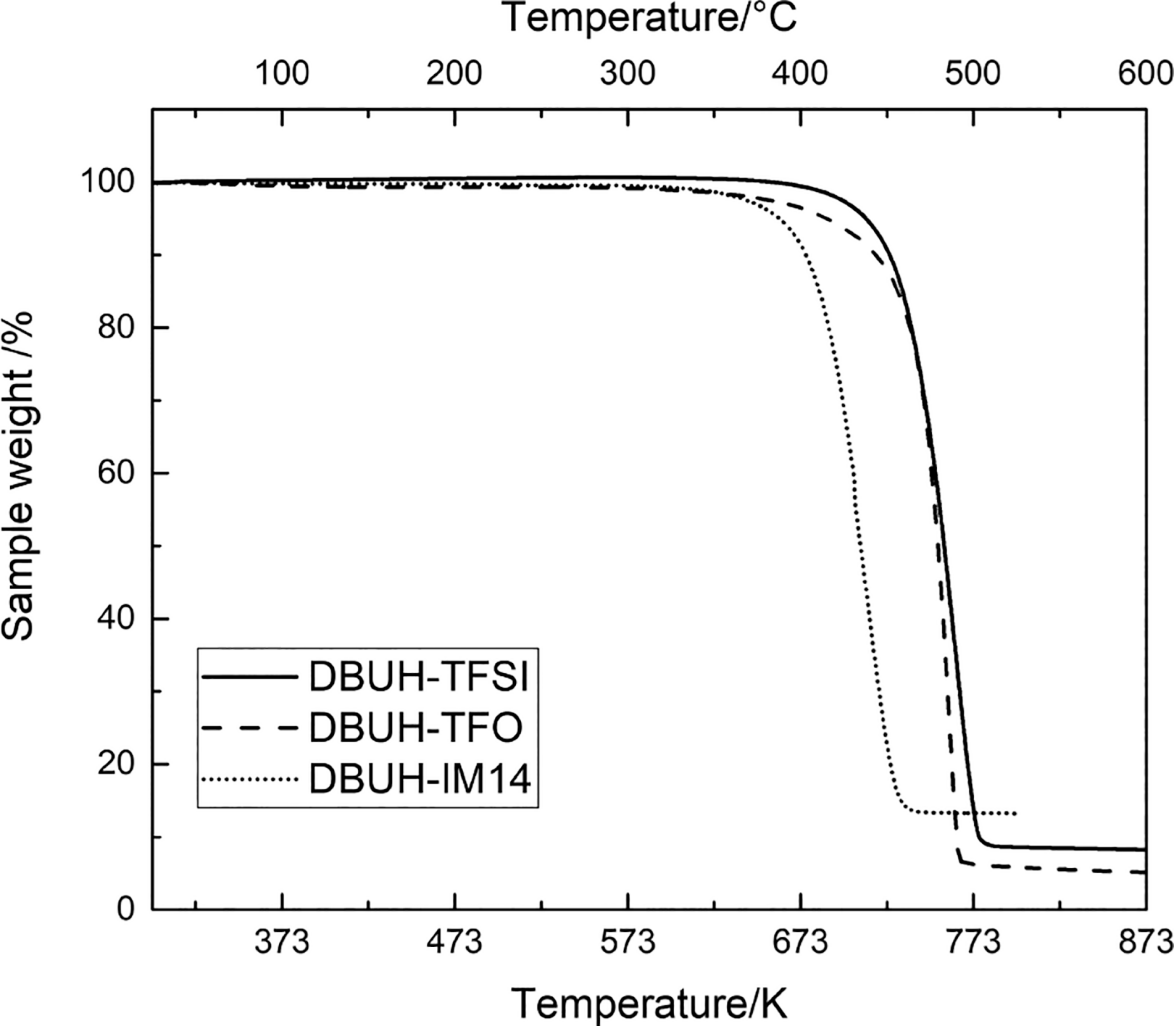
Dynamic TGA curve of the PIL samples in nitrogen. Scan rate: 10 K min^–1^.

### 3.3 Density and Viscosity

The density ρ and viscosity η were measured for the PIL samples as a function of temperature, and experimental values are reported in Tables S3 and S6.

For all the PILs, the density was fitted to a linear equation in the range of temperature evaluated, showing a decrease with increasing temperature (Figure S13 and best fit parameters in Table 3). A graphic comparison of the results obtained here with those available in the literature (Figure S13) for DBUH-IM14 points out that the difference between the experimental and literature data^19^ is lower than the standard uncertainty of the measurement. Conversely, the density values of DBUH-TFO and DBUH-TFSI reported by Miran et al.^14^ show a considerable deviation (0.005 g cm^–3^ at 298 K and around 0.01 g cm^–3^ at 308 K for DBUH-TFO and 0.018 g cm^–3^ at 308 K for DBUH-TFSI) from those of the present work. These differences may stem from a different purification treatment of the PILs because the presence of impurities frequently reduces the density of ionic liquids.^45^

**Table 3:** Linear Fit Parameters of the Density, ρ = ρ_0_ – *aT* (K)

PIL	*T* range (K)	*a* (g cm ^3^ K^–1^)	ρ_0_ (g cm^–3^)	*R*^2^	*M* (g mol^–1^)
DBUH-IM14	298–358	10.7 × 10^–4^	1.898	0.999	583.4
DBUH-TFSI	308–368	9.00 × 10^–4^	1.758	0.999	433.4
DBUH-TFO	298–358	7.92 × 10^–4^	1.586	0.999	302.3

For the DBU-based PILs the densities decreased in the order [IM14^–^] > [TFSI^–^] > [TFO^–^]. This trend agrees with the less structured and less packed DBUH-IM14 when compared to DBUH-TFO, as reported in the literature,^19^ confirming that the nature of the anion influences the density of an ionic liquid.^46^

The isobaric thermal expansivity (α_*p*_) can be calculated from the density as described in the Supporting Information.^47^ At atmospheric pressure, α_*p*_ is monotonically increasing with increasing temperature for all the DBU-based PILs studied (Figure S14 and Table S4). Similar behavior is expected for organic solvents,^48^ and it is also reported for other protic^49^ and aprotic ionic liquids.^50^ A more pronounced temperature dependence is observed for the DBUH-IM14 (Table S5), which contains the largest anion. Indeed, ILs with large molecular weight are reported to behave more like a molecular liquid: the large size of the ion and the charge delocalization tend to dilute the ionic character, thus mimicking the molecular behavior of an organic solvent.^51^ This indirectly suggests that the ionic character of DBUH-IM14 is weaker than that of the other PILs DBUH-TFSI and DBUH-TFO (*vide ultra*).

For all systems the viscosity decreases with increasing temperature (Figure S15). The temperature dependence of the data is described by the Vogel–Fulcher–Tammann (VFT) equation, with best-fit parameters in Table 4. The viscosity trend follows the order: DBUH-TFSI < DBUH-IM14 < DBUH-TFO, with DBUH-IM14 three times less viscous than DBUH-TFO at ambient temperature. Given the viscosity results from the interplay between structural features and molecular interactions,^52^ the highest viscosity of DBUH-TFO can be ascribed to the strong hydrogen bonds between the protonated DBU cation and the triflate.^19^ When comparing instead DBUH-TFSI and DBUH-IM14, which have comparable hydrogen bonding, the viscosity increases with increasing the ion size (e.g., on passing from TFSI^–^ to IM14^–^) reflecting again the key role played by the structural features of the large IM14^–^ anion with asymmetrical fluorine-containing chain organization and its perfluorocarbon domains.^20^

**Table 4:** VFT Fit Parameters of the Viscosity, η = η_0_ exp[*B*/(*T* – *T*_0_)]

PIL	η_0_ (mPa·s)	*B* (K)	*T*_0_ (K)	*R*^2^
DBUH-TFO	0.0604	1200.2	183.5	0.9999
DBUH-TFSI	0.0891	944.2	180.7	0.9999
DBUH-IM14	0.0605	1098.5	183.6	0.9999

### 3.4 Ionic and Molar Conductivities

**Figure 6:**
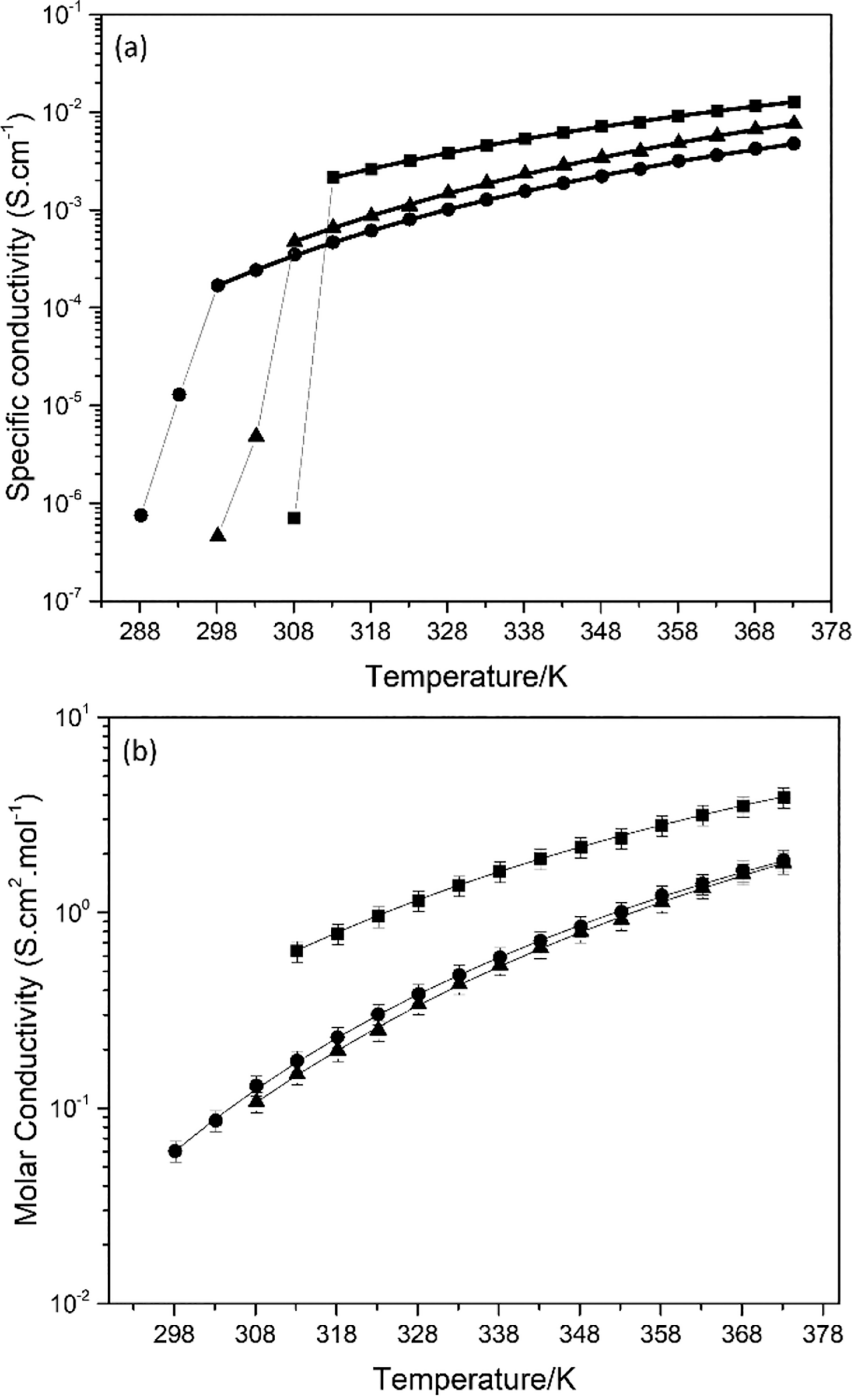
(a) Ionic and (b) molar conductivity of the PILs (■) DBUH-TFSI, (●) DBUH-IM14, and (▲) DBUH-TFO as a function of the temperature. Black solid lines correspond to the VFT fitting.

Given the relevance of high charge transport for electrochemical applications, understanding the features governing the ionic conductivity (σ) of DBUH-IM14 is crucial to optimize its application as electrolyte component. In the present work, the mobility of ionic carriers was investigated by using electrochemical impedance spectroscopy (EIS, Figure 6a and Table S7).

Below the melting point, the DBU-based PILs showed low ionic conductivity in the range of 10^–7^ S cm^–1^. A sharp increase of the specific conductivity indicates the complete melting of the PILs samples. All the systems reported here show good ionic conductivity (in the order of 10^–3^ S cm^–1^ at 333 K) comparable to other PILs reported in the literature.^53^

In the temperature range where the samples are in the liquid state, the temperature dependencies of the ionic conductivity (σ) follow the VFT equation (typical of IL materials)^34^
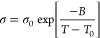
1where σ_0_ (S cm^–1^), *B* (K), and *T*_0_ (K) are adjustable parameters.

The best-fit parameters for the VFT model of the specific conductivity data are listed in Table 5, while the graphical representation of the experimental and fitted data is shown in Figure 6a as black solid lines.

**Table 5:** VFT Fit Parameters of the Ionic Conductivity

PIL	*T* range (K)	σ_0_ (S cm^–1^)	*B* (K)	*T*_0_ (K)	*R*^2^
DBUH-TFSI	313–373	0.50	674.12	189.51	0.999
DBUH-TFO	308–373	1.30	950.27	188.02	0.999
DBUH-IM14	298–373	0.45	806.86	195.77	0.999

When the samples are in the liquid state, their specific conductivity decreases in the order DBUH-TFSI > DBUH-TFO > DBUH-IM14, which is not in line with the fluidity trend DBUH-TFSI > DBUH-IM14 > DBUH-TFO. Hence, lower ionic mobility is detected in the bulk liquid of DBUH-IM14 when compared to DBUH-TFO, despite the higher fluidity of the former. Although DBUH-IM14 shows the lowest specific conductivity among the PILs investigated, this is the only system that provides practical conductivity value extending down to room temperature.

The molar conductivity of the PILs studied (see the Supporting Information for more details) follows the order DBUH-TFSI > DBUH-IM14 ≈ DBUH-TFO (Figure 6b). Although DBUH-IM14 has lower viscosity than DBUH-TFO, both systems show a comparatively high molar conductivity. This latter point seems to suggest that despite DBUH-IM14 showing a softer H-bond network, long-range attractive dispersive interactions among the fluorous tails may be causing a decreasing on its molar conductivity. The net balance provides a comparable molar conductivity in the two cases and a clear indication that the molecular volume, the structure, and the repertoire of interaction of the anions strongly influence the macroscopic property of this class of PILs, in line with what already reported for a series of aprotic ionic liquids.^54^

### 3.5 Ionicity

**Figure 7:**
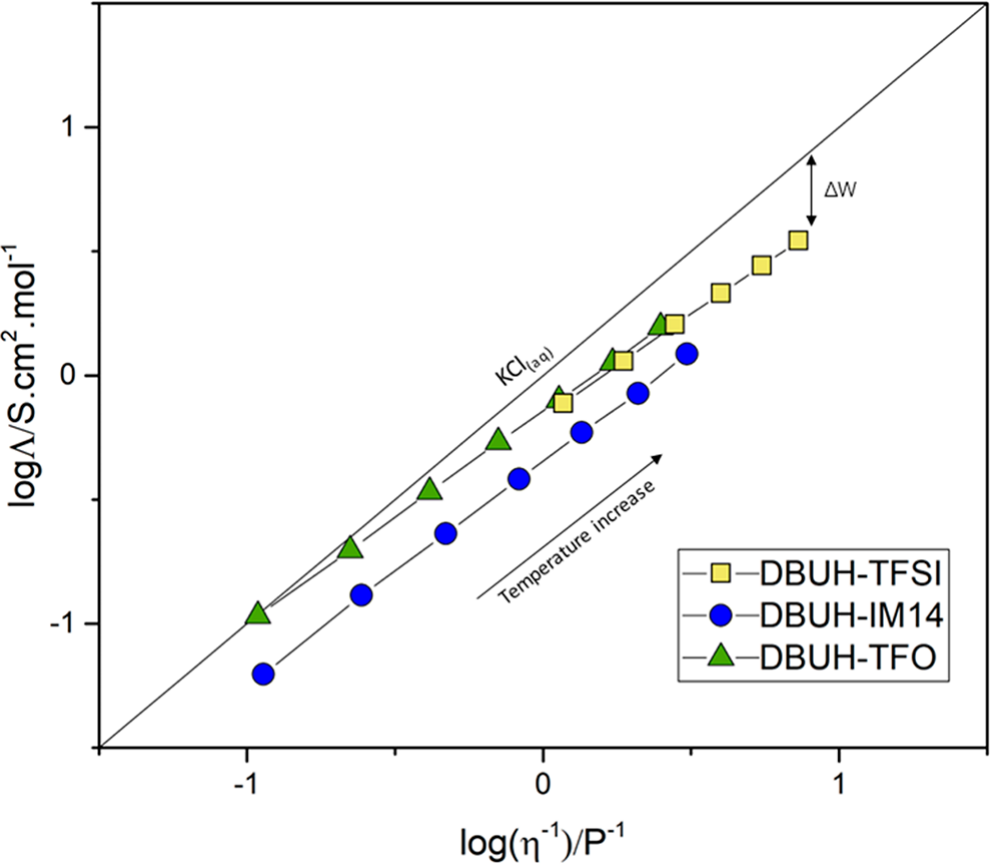
Walden plot of the PILs studied.

In a PIL, the effective fraction of ions participating in the conduction process can be estimated by the ionicity using the readily accessible approach based on the Walden plot (see the Supporting Information).^7^ Ionicity values far below unity evidence the low ionic character of the system, which highly deviates from the behavior of an ideal electrolyte system. The Walden plot of the PILs studied and their calculated values for ionicity are displayed in Figure 7 and Table S10.

DBUH-TFO (0.63 < *I*_w_ < 0.99) and DBUH-TFSI (0.48 < *I*_w_ < 0.66) are more ionic systems than DBUH-IM14 (0.38 < *I*_w_ < 0.55). The ionicity values found for DBUH-TFO are counterintuitive as this PIL has strong hydrogen bonds which are expected to cause strong ion correlations preventing the use of the Walden rule to describe the ionicity in such a viscous system.^55,56^ When comparing DBUH-IM14 with DBUH-TFSI—that is, systems with similar intermolecular interactions (i.e., “soft” hydrogen bonds)—the strong difference between their ionic behavior is quite surprising. This notable discrepancy may be directly associated with the structural features of the IM14^–^ anion. Particularly, the strong nature of like-ion anticorrelations (cation–cation and anion–anion) is known to influence the conductivity of the system lowering its ionicity.^57^ Such anion–anion effects could be more significant in IM14^–^-based PILs due to its geometrical nature, steric hindrance, and the presence of fluorous domains via anion–anion interactions.^19^ Further investigations are currently in progress to support this assumption.

## 4 Conclusions

The physicochemical and transport properties of the novel room temperature PIL DBUH-IM14 were widely investigated and compared with PILs also generated via proton transfer from strong acids to DBU but showing different anion’s structure (DBUH-TFSI and DBUH-TFO). IM14^–^ is a remarkably large anion with an uneven distribution of the C–F pool between the two sides of the sulfonylimide moieties, which impart peculiar structural effects to the corresponding PIL, including spatial segregation of fluorinated tails. ^1^H and ^15^N NMR spectra allowed for the first time the indirect measurement of Δp*K*_a_ of DBUH-IM14 (23.4) and proton affinity of IM14^–^ (294 kcal/mol), which are comparable to DBUH-TFSI (so far among the PILs with the highest proton transfer in the literature). Besides, the N–H bond between the imino nitrogen of the DBU and the acidic proton was shown to remain thermodynamically and kinetically stable in the whole range of temperatures evaluated. In light of the NMR findings of the present work, the H-bond donor species (DBUH) is completely and stably protonated in all the studied systems, thus confirming that the properties of the liquid are modulated by size, shape, and electronic properties of the anions.

The investigation of the thermal properties of the DBUH-IM14 confirmed that it is the only system liquid at room temperature enabling its technical application. In addition, the thermal stability evaluation confirms that in the case of DBUH-IM14 factors such as the conformational space available to the perfluorinated butyl chain, the potential formation of fluorous domains via anion–anion interactions, and the hydrogen bond network in the liquid structure of DBUH-IM14 are factors flanking the acid strength descriptors in the physical chemical characterization. From a practical viewpoint and in perspective applications, the fact that DBUH-IM14 showed the lowest thermal stability of the three PILs investigated should not be overemphasized, as the decomposition of DBUH-IM14 occurs far above 673 K, with a practical weight loss observed only above 523 K. Consequently, DBUH-IM14 can still be considered eligible for high-temperature applications. Noteworthy is the surprisingly good molar conductivity detected for DBUH-IM14 especially at low temperature, which enhances its potential application as electrolyte component. The complex combination and interplay of different interaction types (H bond, Coulomb, and dispersive) in DBUH-IM14 seem indeed to affect its charge transport as revealed by the Walden plot. The extensive characterization given here for the DBUH-IM14 not only is relevant to guide scientists in the future design and application of this PIL but also evidenced a key point of IL research field: the structural features of the PILs’ constituents need to be considered when tailoring their properties for a selected purpose.

## Supporting Information Available

NMR supplementary data, DTG and isothermal TGA curves, density and specific conductivity supplementary data, molar conductivity data (PDF)
