# Supplementary material for: Implications of Anion Structure on Physicochemical Properties of DBU-Based Protic Ionic Liquids
Source: J Phys Chem B. 2022 Aug 30;126(36):7006–14. doi: 10.1021/acs.jpcb.2c02789 (PMC9483912; doi:10.1021/acs.jpcb.2c02789)
Supplement: Supplementary file 1 — jp2c02789_si_001.pdf [file jp2c02789_si_001.pdf]

Supplementary Material to:

Implications of Anion Structure on Physicochemical Properties of DBU-Based Protic Ionic Liquids

Giselle de Araujo Lima e Souza,[a] *Maria Enrica di Pietro,*[*a] *Franca Castiglione,*[a] *Pedro Henrique Marques Mezencio,*[b] *Patricia Fazzio Martins Martinez,*[b] *Alessandro Mariani,*[c,d,e] *Hanno Maria Schütz,*[d,e] *Stefano Passerini,*[d,e] *Maleen Middendorf,*[f] *Monika Schönhoff,*[f] *Alessandro Triolo,*[g] *Giovanni Battista Appetecchi,*[h] *Andrea Mele.*[a]

a. Department of Chemistry, Materials and Chemical Engineering "Giulio Natta", Politecnico di Milano, Piazza Leonardo da Vinci 32, 20133, Milan, Italy.

b. School of Chemical Engineering, University of Campinas, Street Albert Einstein 500, 13083-852, Campinas, Brazil.

c. Università Politecnica Delle Marche, Piazza Roma, 22, 60121 Ancona, Italy

d. Helmholtz Institute Ulm (HIU), Helmholtzstraße 11, D-89081, Ulm, Germany

e. Karlsruhe Institute of Technology (KIT), P.O. Box 3640, D-76021 Karlsruhe, Germany.

f. Institute of Physical Chemistry, University of Muenster, Corrensstrasse 28-30, 48149 Münster, Germany.

g. Istituto Struttura della Materia (ISM), Consiglio Nazionale delle Ricerche (CNR), via Fosso del Cavaliere 100, 00133 Rome, Italy.

h. ENEA (Italian National Agency for New Technologies, Energy and Sustainable Economic Development), Department for Sustainability (SSPT), Casaccia Research Center, Via Anguillarese 301, 00123 Rome, Italy.

Table of Contents

Sample preparation

Synthesis of the PIL sample S3

Purification of the PIL samples S3

NMR measurements

^1H, ^{13}C and ^{15}N NMR experimental details S4

^1H NMR spectra S8

^{13}C-{^1H} NMR spectra S9

1D ^{15}N NMR spectra S10

^{15}N chemical shift as a function of temperature S11

^{15}N INEPT spectra S12

Thermal properties

DSC analysis S14

DSC traces S15

TGA analysis S15

TGA curves S16

DTG curves S17

Isothermal TGA S19

Density and viscosity measurements

Density data and fitting S20

Viscosity data and fitting S23

Electrochemical measurements

Specific conductivity S25

Molar Conductivity S26

Ionicity S28

References S29

Sample preparation

Synthesis of the PIL samples

1,8-diazabicyclo[5.4.0]undec-7-ene (DBU, 98%), trifluoromethylsulfonic acid (HTFO, 99%), and nitric acid (HNO_3, 65 wt.% solution in water) were purchased from Sigma-Aldrich. Lithium bis(trifluoromethanesulfonyl)imide salt (LiTFSI, > 99.9 wt.%) and acidic (trifluoromethanesulfonyl)(nonafluorobutanesulfonyl)imide (HIM14, 60 wt.% solution in water) were provided by 3M. The synthesis of DBUH-TFO and DBUH-IM14 consisted of a standard neutralization reaction. The acid was counted in 3 wt.% excess to assure the full protonation of the DBU. The acid was added dropwise to the base under stirring, kept in a cooling water/ice bath to avoid sudden temperature increase and unexpected oxidation. The DBUH-TFSI was synthesized by reacting DBU with nitric acid for neutralization, yielding DBUH-NO_3. Then, DBUH-TFSI was formed by a metathesis reaction using LiTFSI dissolved in water. The crude DBUH-based ILs were rinsed several times with deionized water to remove water-soluble impurities, acid excess (DBUH-TFO and DBUH-IM14) and, for DBUH-TFSI, water-soluble $LiNO_3$ and the excess of LiTFSI. Finally, all PILs were vacuum dried at 323 K for at least 1 hour and then, at 373 K for at least 18 hours and stored in an Ar atmosphere glove box (VAC, [O_2] < 1 ppm, [H_2O] < 1 ppm).

Purification of the PILs samples

The dry PILs samples were submitted to an additional purification procedure [1] using activated charcoal (Sigma-Aldrich Darco-G60), previously cleaned through a protocol reported elsewhere [2], and ethyl acetate as solvent. The mixture was stirred for at least 3 hours and vacuum filtered. The solvent ethyl acetate was evaporated in a desiccator under vacuum. Finally, the PILs were vacuum dried (< 1 mbar in oil-free pump) at 323 K for at

least 1 hour and then, at 373 K for at least 18 hours. The purified PILs were stored in an Ar atmosphere glove box (VAC, [O_2] < 1 ppm, [H_2O] < 1 ppm). The water content of the final PILs samples, 180 ppm, was measured by Karl-Fischer titration. The 1:1 cation:anion stoichiometry was confirmed by quantitative ^{13}C-{^{1}H} NMR (*vide infra*)

NMR measurements

^{1}H, ^{13}C and ^{15}N NMR experimental details

The PILs samples were placed in 5 mm NMR tubes and flame-sealed in a controlled environment (dry-room, relative humidity < 0.1% at 293 K) to prevent any contamination and air contact. All the NMR tubes contained a flame-sealed capillary tube of deuterated DMSO (DMSO-d_6) as a reference/lock solution.

^{1}H, ^{13}C-{^{1}H} and ^{15}N NMR experiments of the pure PILs were performed at 11.74 T with a Bruker NEO 500 console equipped with a direct observe 5 mm pulsed-field z-gradient BBFO iProbe. For each sample, the probe was carefully tuned, and the signal following 90° pulses was evaluated. The sample temperature was set and controlled using a variable temperature control unit using air gas flow. Standard Bruker library programs were used for the measurements.

Quantitative ^{13}C-{^{1}H} NMR spectra were acquired at 308 K using the inverse-gated decoupling pulse sequence, a repetition delay of 90s and 128 scans for each experiment. FID were submitted to an exponential multiplication with a 20Hz line broadening, automatic phase and baseline correction. Integrals of selected isolated carbons of DBUH cation and the given anion confirmed the 1:1 stoichiometry for all systems.

^{1}H and ^{15}N NMR spectra were acquired over a temperature range from 298 K to 373 K for DBUH-TFO and DBUH–IM14, and from 313 K to 373 K for DBUH-TFSI, with steps

of 5 K, 10 K, or 15 K depending on the type of analysis, and a minimum of 30 minutes allowed for thermal equilibration.

Shortly before the ^{15}N spectra were collected, the PILs were externally referenced to the standard sample of 90% formamide in DMSO-d_6 by setting its ^{15}N resonance to 112.7 ppm. The ^{15}N spectra were collected with and without ^{1}H decoupling, the latter allowing the direct observation of the one-bond spin-spin coupling ($^{1}J_{N\text{-}H}$). Experimental details are reported in the Supplementary Material, Tables S1-S2.

The Insensitive Nuclei Enhancement by Polarization Transfer (INEPT) NMR pulse sequence was applied to allow a faster and more accurate measurement of the $^{1}J_{N\text{-}H}$ over the whole temperature range. INEPT NMR is a useful pulse sequence to increase the sensitivity of the rare nucleus, such as ^{15}N (natural abundance 0.364%) by transferring magnetization from the reservoir of the most abundant nucleus, such as ^{1}H, thus allowing the relatively fast observation of the N-H coupling constant in samples at natural ^{15}N abundance.

Table S1. Experimental details of the ^{15}N-{^{1}H} NMR spectra recorded at 308 K and displayed in Figure 3 of the manuscript.

DBUH-TFSI	
Parameter	Value
NS	178
TD	32768
SW (ppm)	70.46
D1 (s)	10.0
DBUH-IM14	
Parameter	Value
NS	4096
TD	8192
SW (ppm)	147.24
D1 (s)	2.0
DBUH-TFO	
Parameter	Value
NS	96
TD	32768
SW (ppm)	70.46
D1 (s)	10.0
DBU (free base)	
Parameter	Value
NS	256
TD	32768
SW (ppm)	246.62
D1 (s)	10.0

NS: number of scans; TD: time domain - number of raw data points; SW: spectral width; D1: relaxation delay between scans.

Table S2. Experimental details of the ^{15}N NMR coupled spectra acquired at 318 K for DBUH-TFSI, and 305 K for DBUH-IM14, and DBUH-TFO, and displayed in Figure 4 of the manuscript.

DBUH-TFSI	
Parameter	Value
NS	13312
TD	16384
SW (ppm)	147.24
D1 (s)	3.0
DBUH-IM14	
Parameter	Value
NS	13312
TD	16384
SW (ppm)	147.24
D1 (s)	3.0
DBUH-TFO	
Parameter	Value
NS	3000
TD	32768
SW (ppm)	70.46
D1 (s)	10.0

NS: number of scans; TD: time domain - number of raw data points; SW: spectral width; D1: relaxation delay between scans.

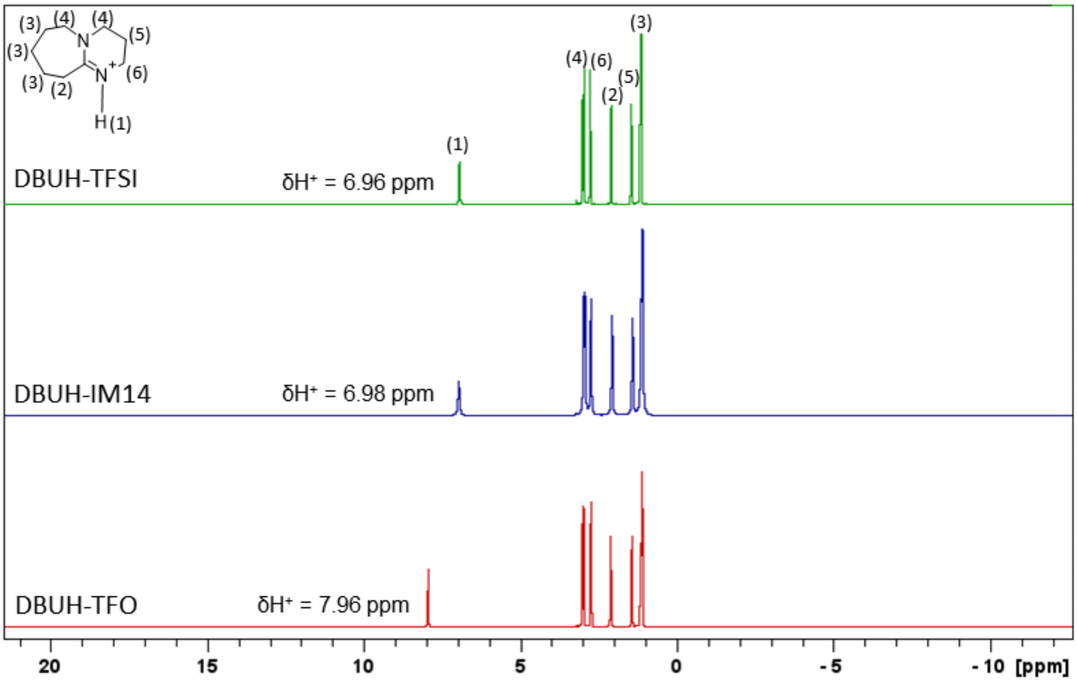

Figure S1. ^{1}H NMR spectra and chemical shift assignment of the PILs investigated at 328 K with spectral width up to 20 ppm.

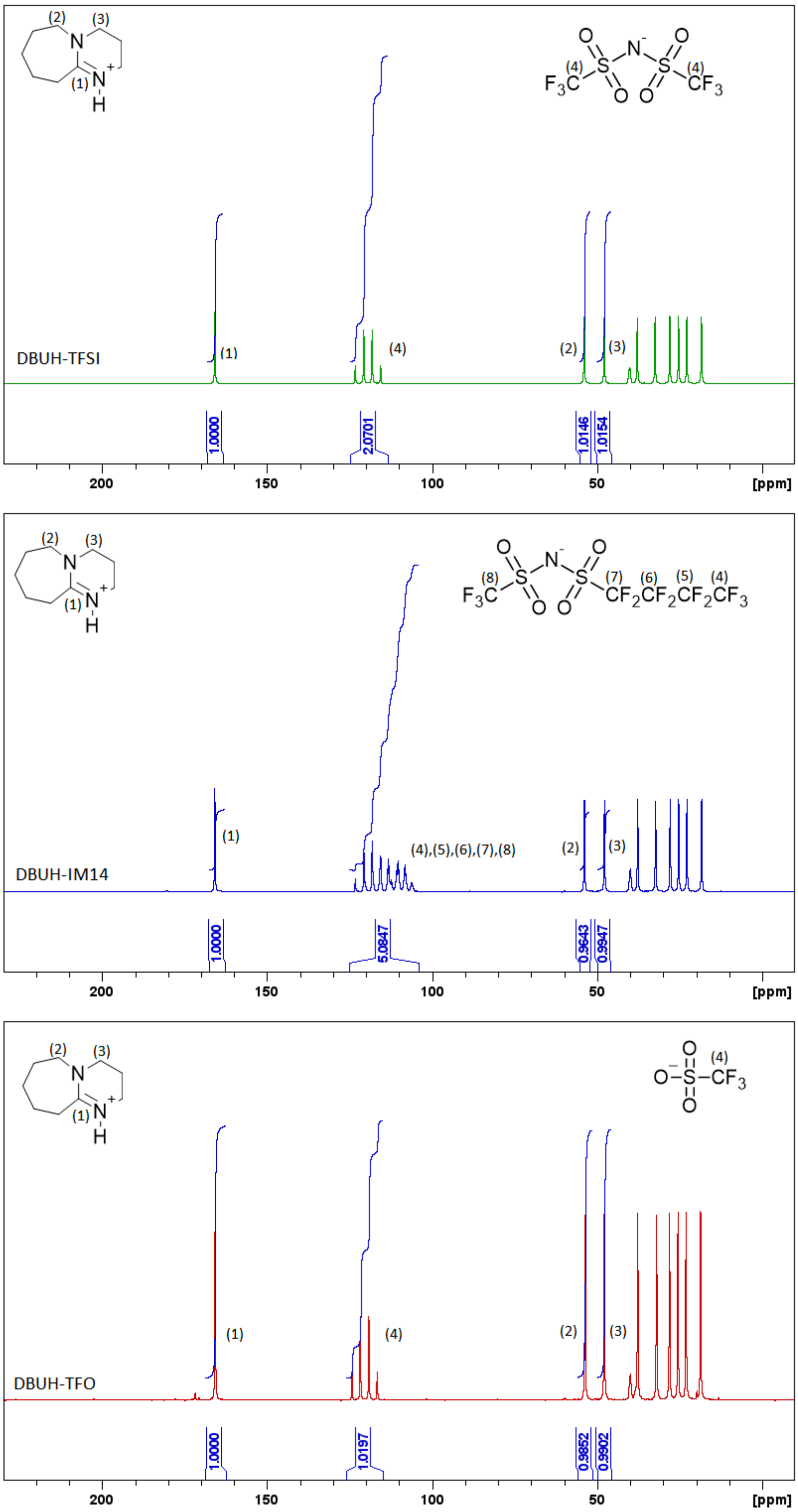

Figure S2. Quantitative ^{13}C-{^{1}H} NMR spectra acquired at 308K for the PILs investigated and integrals of selected isolated signals.

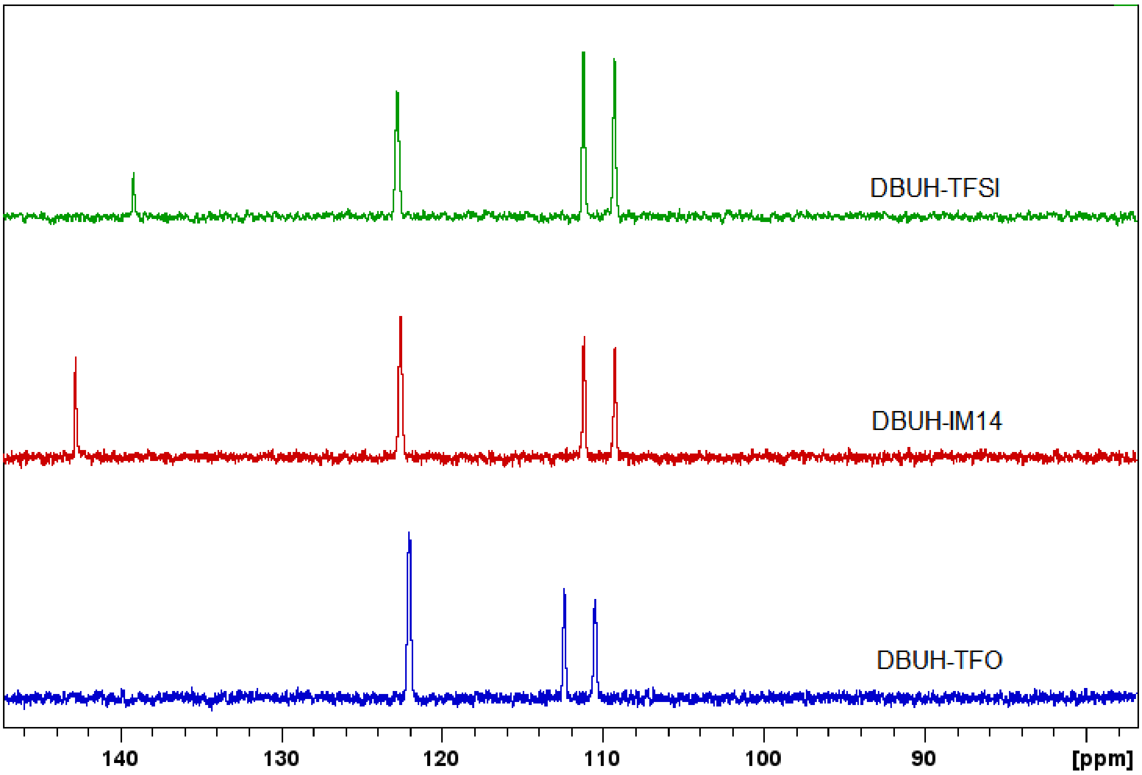

Figure S3. 1D ^{15}N NMR spectra acquired without proton decoupling at 318 K for DBUH-TFSI, and 305 K for DBUH-IM14, and DBUH-TFO.

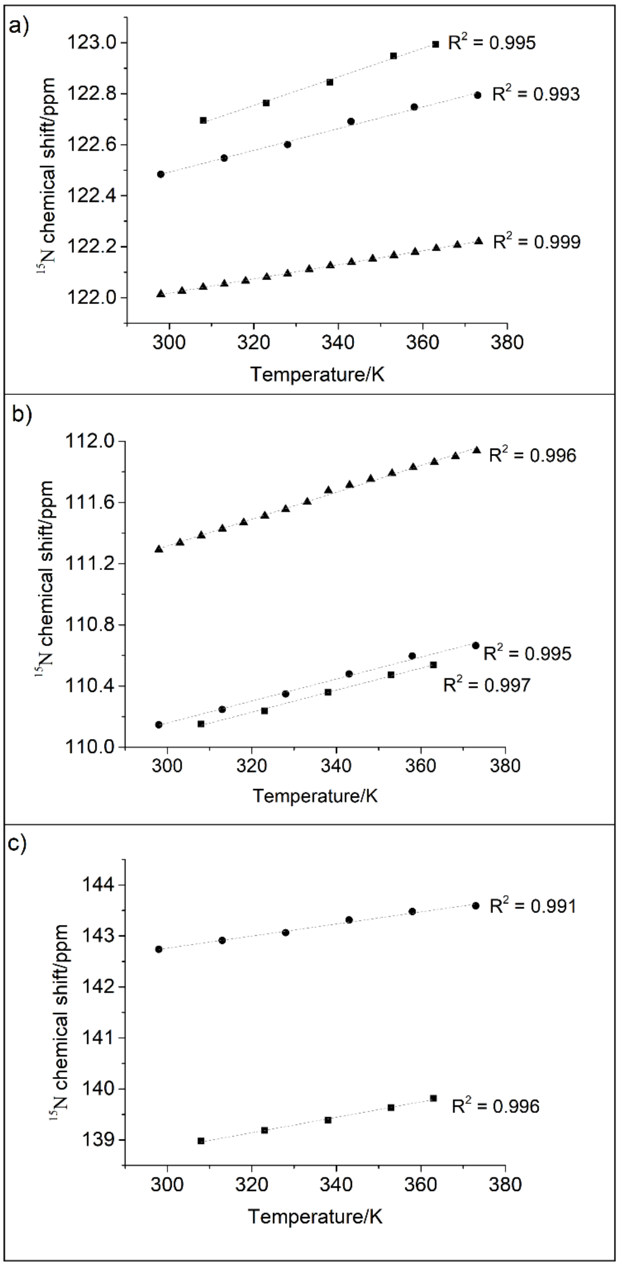

Figure S4. ^{15}N chemical shift of the (a) imino (N2), (b) amino (N1) and (c) imide nitrogen for the (■) DBUH-TFSI (●) DBUH-IM14 and (▲) DBUH-TFO, as measured from ^{15}N-{^{1}H} NMR spectra as a function of temperature.

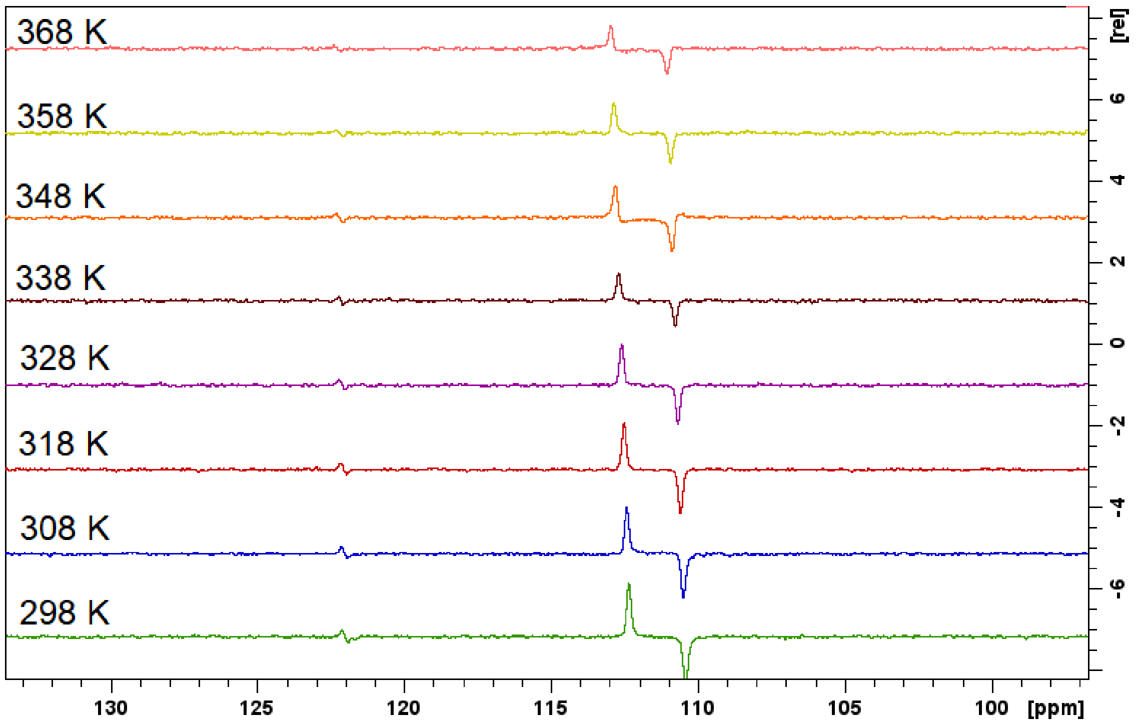

Figure S5. ^{15}N INEPT spectra recorded for DBUH-TFO as a function of temperature.

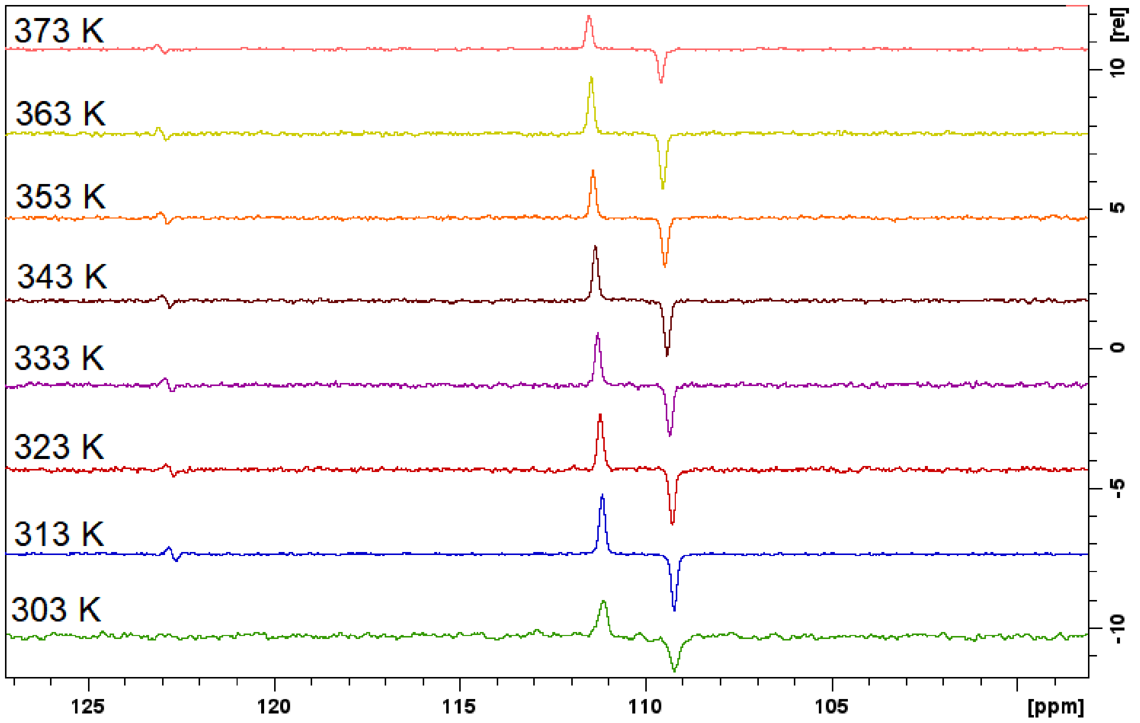

Figure S6. ^{15}N INEPT spectra recorded for DBUH-TFSI as a function of temperature.

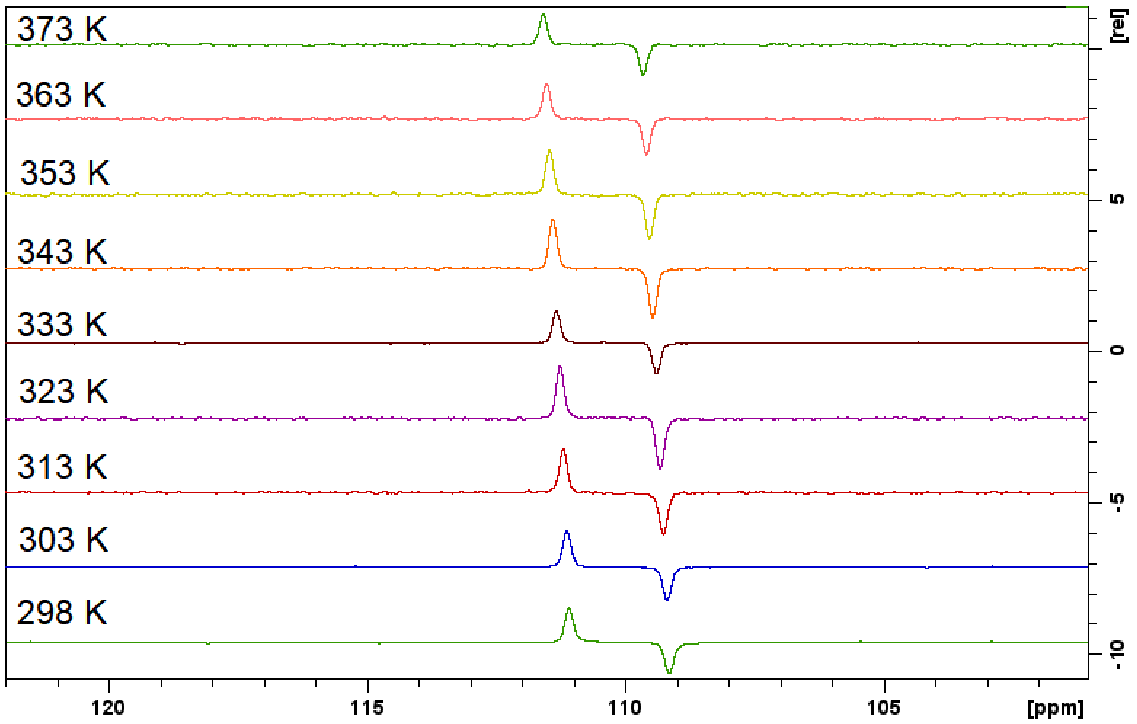

Figure S7. ^{15}N INEPT spectra recorded for DBUH-IM14 as a function of temperature.

Thermal properties

DSC analysis

Thermal properties of the PILs samples were analyzed by differential scanning calorimetry (DSC), using the instrument DSC 1 (Mettler Toledo), equipped with a liquid nitrogen cooling system. About 10 mg of the PILs samples were weighted using the microanalytical balance MX5 (Mettler Toledo) and tightly sealed in standard aluminum crucible. The liquid samples, DBUH-IM14 and DBUH-TFO, were subjected to the same temperature program. First, the samples were cooled down to 153 K, with isothermal treatment at this temperature for 2 minutes. Then, they were heated up to 323 K. The heating and cooling scan rates were 10 $K.min^{-1}$. An additional evaluation was performed on both samples, fully crystallizing them before the heating scan. Thus, the PILs were subjected to a refrigeration cycle from 153 K until their cold-crystallization temperature in the DSC instrument [3]. Then, the thermograms were recorded during the heating scan from 153 K to 323 K at 10 $K.min^{-1}$.

For the DBUH-TFSI sample, solid at room temperature, the protocol was slightly different. The sample was initially heated from 298 K to 323 K, at 10 $K.min^{-1}$, with isothermal treatment at this temperature for 2 minutes. Then, the sample was cooled to 153 K at 2 $K.min^{-1}$ and kept at 153 K for 5 minutes, for the crystallization process. Finally, the sample was heated to 323 K at a scan rate of 2 $K.min^{-1}$. The DSC thermograms were recorded during the last heating scan.

In the present work, the melting point (T_m) was determined as the extrapolated onset temperatures of an endothermic peak during the heating (or reheating) scan. The cold crystallization temperature ($T_{c\text{-}c}$) was defined as the onset of an exothermic peak upon heating from a subcooled liquid state to a crystalline solid phase. The glass transition temperature (T_g) was obtained as the midpoint of a small heat capacity change upon

heating (or reheating) from the amorphous glass state to a liquid phase. The procedure to establish the thermal events in ionic liquids is well described in the literature [4].

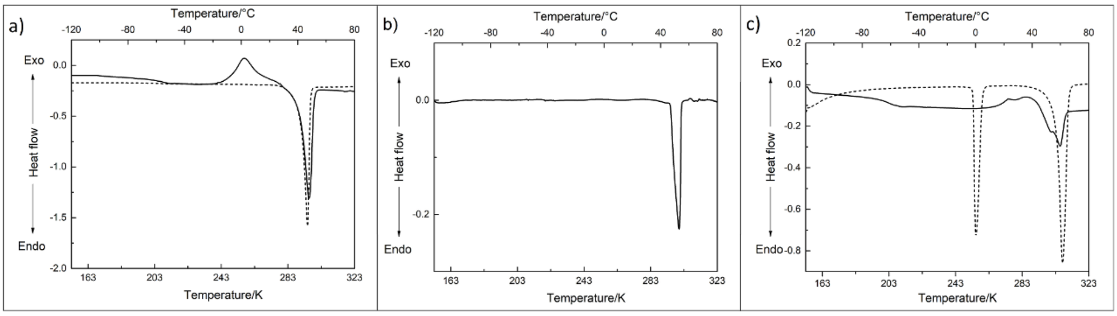

Figure S8. DSC traces of the studied PILs. a) DBUH-IM14, b) DBUH-TFSI, c) DBUH-TFO. For the plots (a) and (c) solid lines are the first heating scan and the dotted lines are the last heating scan after 3 full heating-cooling cycles.

TGA analysis

The thermal stability of the three samples was assessed in nitrogen and synthetic air by thermogravimetric analysis (TGA) using the instrument TGA/DSC1 from Mettler Toledo with the STARe Thermal Analysis Software 10.00. During the experiment, the gas flow rate was settled at 100 $ml.min^{-1}$ to avoid external contamination from the atmosphere. Then, open alumina crucibles containing 10–15 mg of PIL were scanned. Initially, the thermal stability was investigated by heating the samples from room temperature up to 873 K at the heating scan of 10 $K.min^{-1}$.

The decomposition temperature (T_d) is frequently defined as the onset temperature [5] or the temperature where the mass loss is 3%. However, a better approach is based on to estimate the T_d taking the minimum of the TGA derivative curve (DTG) [6]. In the present work, the derivative method (DTG) was applied to determine the decomposition temperatures of the PILs studied. Besides, the samples were evaluated by isothermal TGA

in synthetic air using different temperature steps to obtain more profound information on the stability at higher temperatures.

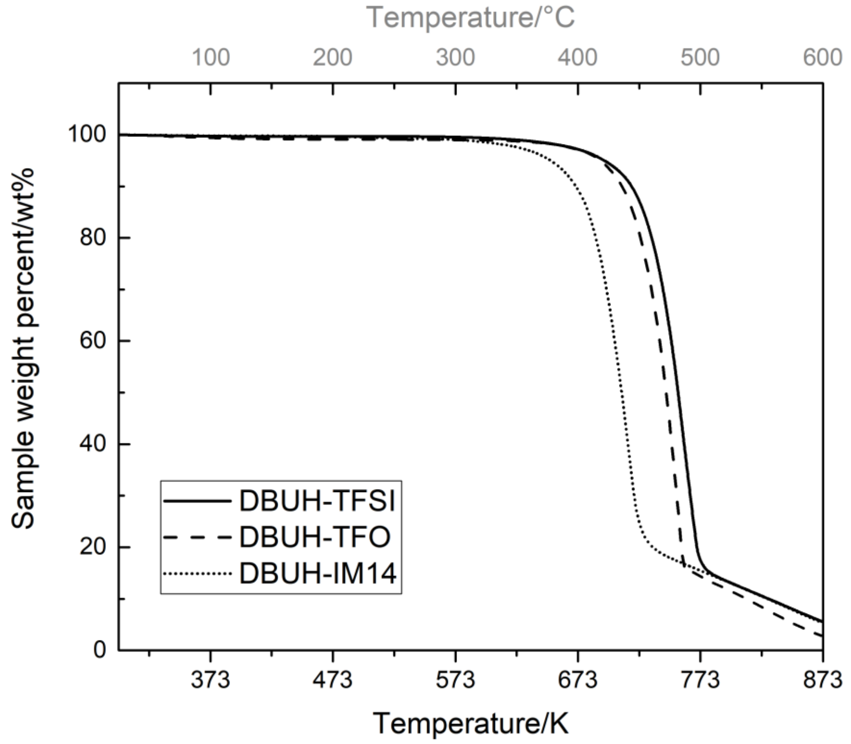

Figure S9. Dynamic TGA curve of the PIL samples in synthetic air. Scan rate 10 K.min^{-1}

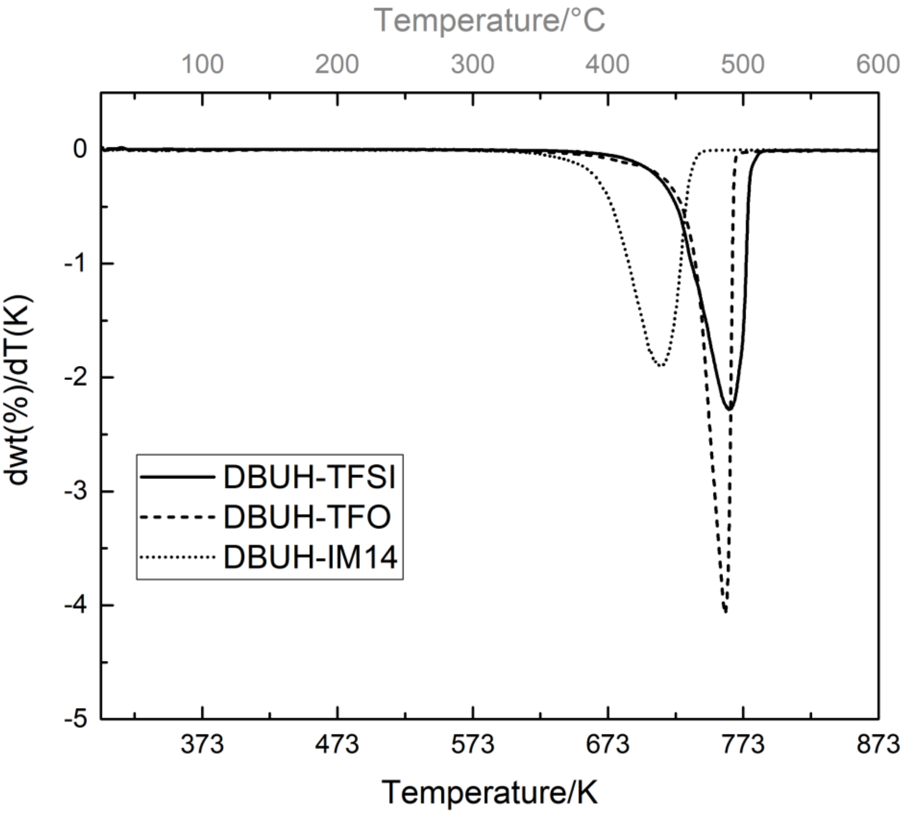

Figure S10. DTG curve of the PIL samples in nitrogen. Scan rate: 10 K.min^{-1}.

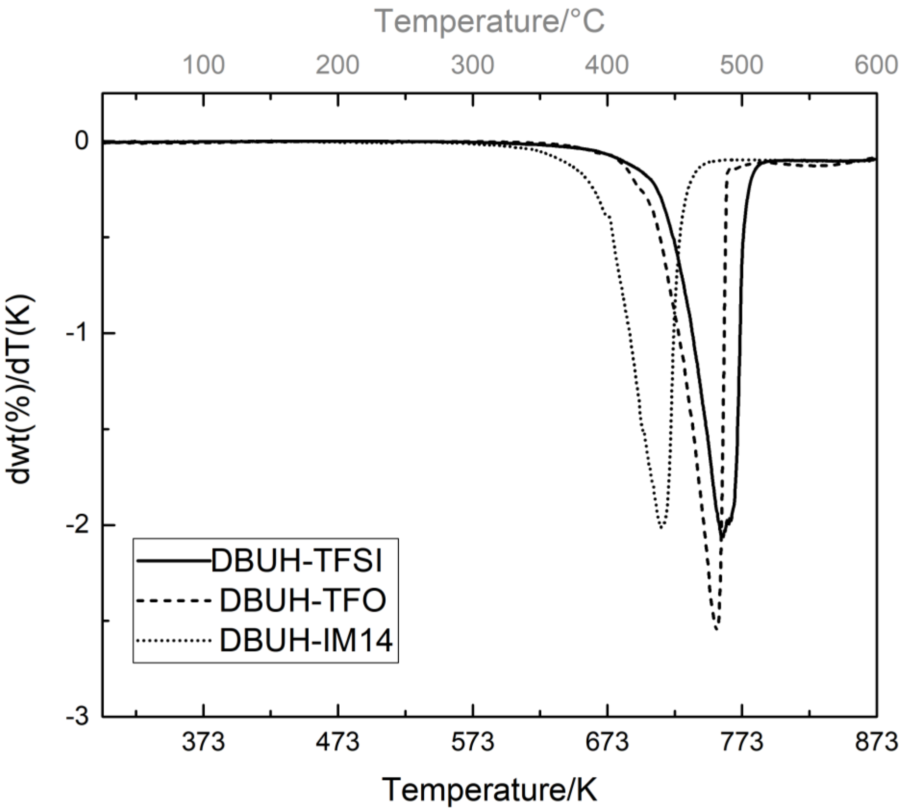

Figure S11. DTG curve of the PIL samples in synthetic air. Scan rate: 10 $K.min^{-1}$.

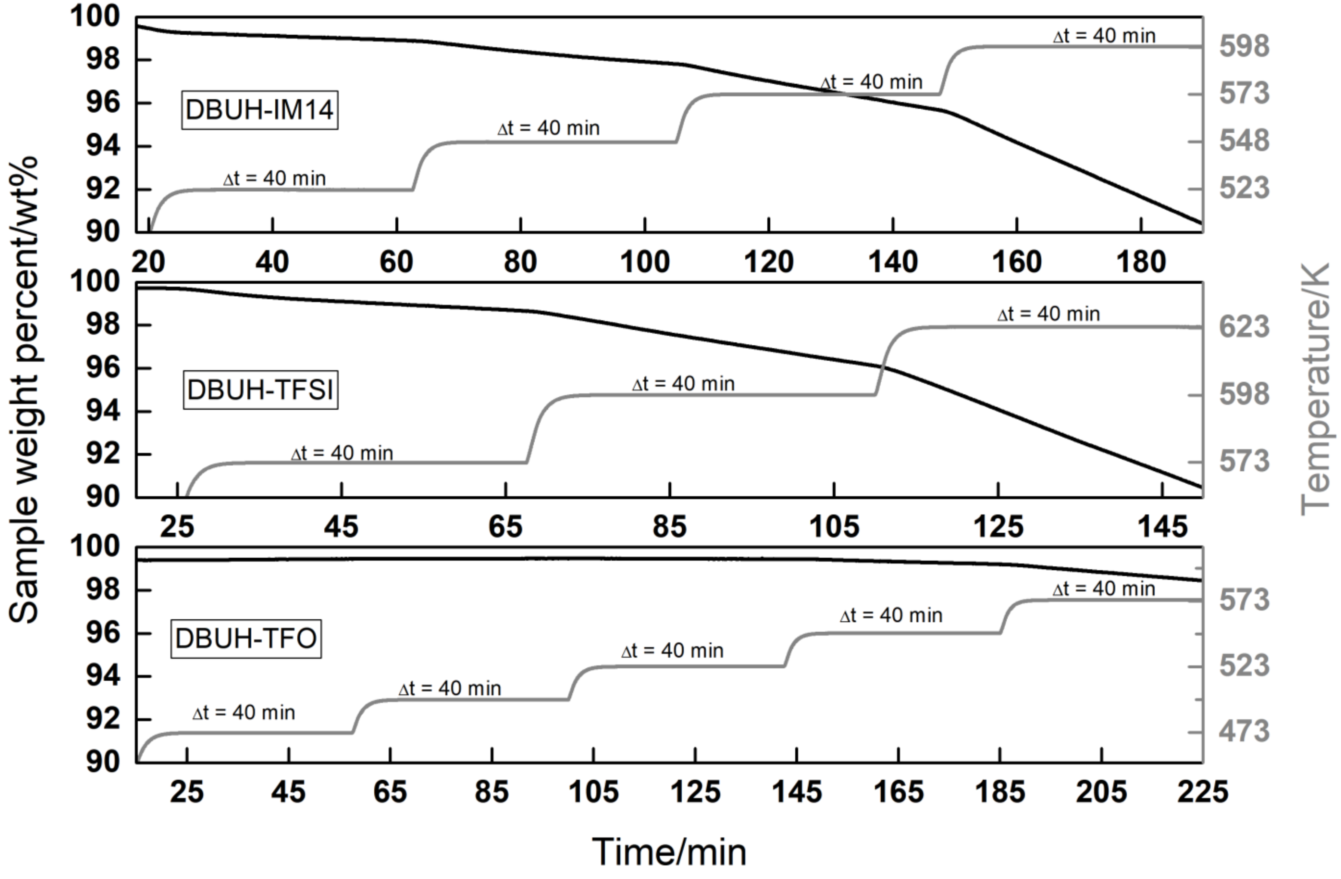

Figure S12. Isothermal TGA of the PIL samples in synthetic air.

Density and viscosity measurements

Density data and fitting

Density measurements of DBUH-TFSI were performed inside an Ar atmosphere glove box (VAC, $[O_2]$ < 1 ppm, $[H_2O]$ < 1 ppm) because the material is highly hygroscopic. There, densities (ρ) were recorded using the density meter DDM2910 from Rudolph Research Analytical in 5 K steps. The other two samples, DBUH-IM14 and DBUH-TFO, were externally manipulated without a controlled environment and the air exposure was minimized as much as possible. The densities of those two samples were measured using the density meter DM45 Delta Range from Mettler Toledo in 10 K steps. For all PILs, the samples were allowed to equilibrate for at least 15 minutes before the measurements.

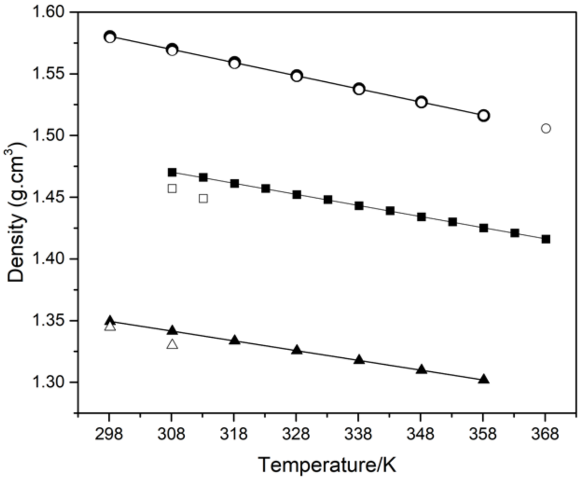

Figure S13. Densities of the PILs (■) DBUH-TFSI (●) DBUH-IM14 and (▲) DBUH-TFO, as a function of temperature. The values for (□)DBUH-TFSI (○) DBUH-IM14 and (Δ) DBUH-TFO correspond to the values fitted from the literature [14,]. Standard uncertainties for the temperature and densities are 0.05 K and 0.001 g.cm^{-3}, respectively.

Table S3. Densities values of the PIL samples at 0.1 MPa.*

	DBUH-IM14	DBUH-TFO		DBUH-TFSI
Temperature (K)	Density ($g.cm^{-3}$)	Density ($g.cm^{-3}$)	Temperature (K)	Density ($g.cm^{-3}$)
298.15	1.580	1.349	308.15	1.470
308.15	1.570	1.341	313.15	1.466
318.15	1.559	1.334	318.15	1.461
328.15	1.548	1.326	323.15	1.457
338.15	1.538	1.318	328.15	1.452
348.15	1.527	1.310	333.15	1.448
358.15	1.516	1.302	338.15	1.443
			343.15	1.439
			348.15	1.434
			353.15	1.430
			358.15	1.425
			363.15	1.421
			368.15	1.416

* Standard uncertainties are 0.05 K and 0.001 $g.cm^{-3}$ for the temperature and densities, respectively.

The calculation of the isobaric thermal expansivity (α_p), leads to useful information on the dependence of the volumetric properties on temperature. To calculate α_p, first, a second-order polynomial function for the temperature dependence of the ln(ρ) was chosen (Eq. S1)[7,8]:

$$\ln\left(\frac{\rho}{\rho_0}\right) = A + B \cdot (T) + C \cdot (T)^2 \qquad \text{(S1)}$$

where ρ is the experimental density at each pressure and temperature; ρ_0 is assumed to be 1.0 $kg.m^3$; T is the temperature; and A, B and C are constant parameters determined from the experimental data using a second-order polynomial

The fitting parameters obtained allow for the calculation of the corresponding α_p values at constant pressure (p) (Eq. S2)[7,8]

$$\alpha_p = -\left(\frac{\partial ln\rho}{\partial T(K)}\right)_P = -[B + 2C(T)] \qquad \text{(S2)}$$

The A, B and C parameters for each PIL are reported in Table S4 and the temperature dependent isobaric thermal expansion coefficient, (α_P) at ambient pressure P is shown in Figure S14. Overall, α_P increases with the increase of the anion size, similarly to other class of ionic liquids[9,10]. As shown in Fig. S13, α_P is linearly dependent on temperature. Comparing the slope of these linear trends (Table S5) shows that DBUH-IM14 is more affected by the temperature, followed by the DBUH-TFSI and finally DBUH-TFO.

Table S4: First order polynomial fitting parameters for density and isobaric thermal expansion coefficient for the studied PILs.

PIL	T range (K)	A	$B \cdot 10^4/K^{-1}$	$C \cdot 10^7/K^{-2}$	R^2	$\alpha_p \cdot 10^4$ range/K^{-1}
DBUH-IM14	298-358	7.516	-3.53	-5.11	0.9999	6.58-7.19
DBUH-TFSI	308-368	7.452	-4.27	-2.91	0.9998	6.06-6.41
DBUH-TFO	298-358	7.367	-4.84	-1.73	1	5.87-6.08

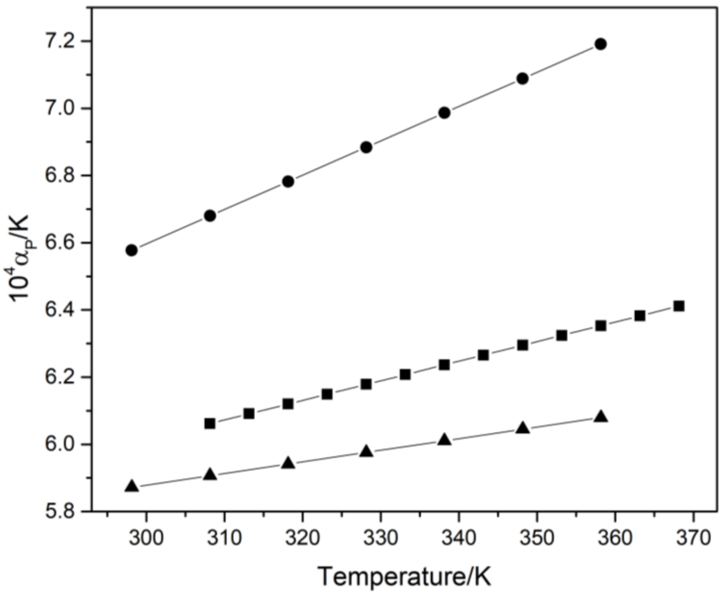

Figure S14. Isobaric thermal expansion coefficient at 0.1 MPa of the PILs (■) DBUH-TFSI (●), DBUH-IM14, and (▲) DBUH-TFO.

Table S5. Linear fitting parameters of the isobaric thermal expansion coefficient (α_P) as a function of temperature (T).

$\propto_P = A + m \cdot T$			
PIL	A	m	R^2
DBUH-IM14	$4 \cdot 10^{-4}$	$1 \cdot 10^{-6}0$	1
DBUH-TFSI	$4 \cdot 10^{-4}$	$0.6 \cdot 10^{-6}$	1
DBUH-TFO	$5 \cdot 10^{-4}$	$0.3 \cdot 10^{-6}$	1

Viscosity data and fitting

The viscosity measurements were carried out in a controlled environment (dry-room, relative humidity < 0.1% at 293 K) to prevent any contamination and air contact. The measurements were performed using a MCR 102 Rheometer (Anton Paar) in a plate-plate geometry (PP25/DI/Ti rotor) with a 100 µm gap. The temperature was controlled using a Peltier-heated P-PTD200/DI base plate and an H-PTD200 actively heated geometry housing. The viscosities values were measured from the loading temperature (i.e., 293 K for DBUH-IM14 and DBUH-TFO and 303 K for DBUH-TFSI, up 373 K with a 5 K temperature step. Each temperature was held for 10 minutes for thermal equilibration, and the viscosity was recorded with a constant shear rate of 10 s^{-1}. All the measurements were performed in triplicates. The temperature-dependence of the data can be described by the Vogel–Fulcher–Tammann:

$$\eta = \eta_o exp\left[\frac{B}{T - T_0}\right] \tag{S3}$$

where η_o (mPa.s), B (K), and T_0 (K) are adjustable parameters.

Table S6. Viscosity data of the studied PILs.

	Viscosity (mPa.s)		
Temperature (K)	DBUH-IM14	DBUH-TFO	DBUH-TFSI
293.15	1364	3417	
298.15	881	2118	
303.15	592	1369	201
308.15	410	919	147
313.15	292	633	111
318.15	213	448	85
323.15	159	326	67
328.15	120	242	53
333.15	93	184	44
338.15	74	142	36
343.15	59	111	31
348.15	48	88	25
353.15	39	71	21
358.15	33	58	18
363.15	27	48	16
368.15	23	40	14
373.15	20	34	12

*Standard uncertainty of the viscosity is 8%; standard uncertainty of the temperature is 0.5 K.

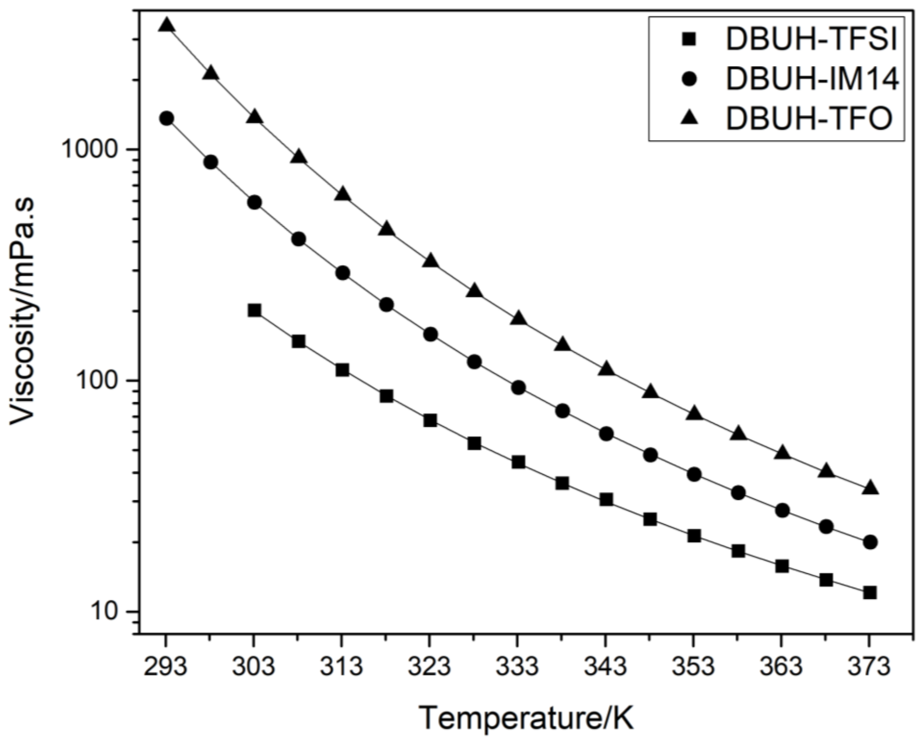

Figure S15. Viscosity of the PILs studied. Solid lines correspond to the VFT fitting.

Electrochemical measurements

Specific conductivity

The specific conductivity was determined by impedance spectroscopy using the potentiostat Parstat 2273. The complex impedance spectra were recorded in the frequency range of 100 kHz to 1 Hz. Before the measurements, the PILs samples were housed in sealed glass conductivity cells equipped with two porous platinum electrodes under an Ar atmosphere in a dry glove box (VAC, $[O_2]$ < 1 ppm, $[H_2O]$ < 1 ppm). In sequence, the cells were dipped in liquid nitrogen to crystallize the PILs. Then, they were kept in the climatic chamber at 233 K for 18 hours, allowing the sample to thermally equilibrate. After that, the conductivity was measured in 5 K steps by running a heating scan at 1 $K.h^{-1}$ from 233 K to 373 K. The specific conductivity (σ) was determined by resolving the Nyquist plots of the impedance data. The uncertainty for the measured specific conductivity is 10%.

Table S7. Specific conductivity of the PIL samples*

	Specific conductivity ($S.cm^{-1}$)		
Temperature (K)	DBUH-IM14	DBUH-TFO	DBUH-TFSI
288.15	$7.51 \cdot 10^{-7}$		
293.15	$1.29 \cdot 10^{-5}$		
298.15	$1.69 \cdot 10^{-4}$	$4.61 \cdot 10^{-7}$	
303.15	$2.42 \cdot 10^{-4}$	$4.81 \cdot 10^{-6}$	
308.15	$3.50 \cdot 10^{-4}$	$4.78 \cdot 10^{-4}$	$7.03 \cdot 10^{-7}$
313.15	$4.68 \cdot 10^{-4}$	$6.61 \cdot 10^{-4}$	$2.15 \cdot 10^{-3}$
318.15	$6.15 \cdot 10^{-4}$	$8.71 \cdot 10^{-4}$	$2.62 \cdot 10^{-3}$
323.15	$8.02 \cdot 10^{-4}$	$1.10 \cdot 10^{-3}$	$3.21 \cdot 10^{-3}$
328.15	$1.01 \cdot 10^{-3}$	$1.49 \cdot 10^{-3}$	$3.85 \cdot 10^{-3}$
333.15	$1.27 \cdot 10^{-3}$	$1.89 \cdot 10^{-3}$	$4.58 \cdot 10^{-3}$
338.15	$1.55 \cdot 10^{-3}$	$2.35 \cdot 10^{-3}$	$5.38 \cdot 10^{-3}$
343.15	$1.88 \cdot 10^{-3}$	$2.87 \cdot 10^{-3}$	$6.23 \cdot 10^{-3}$
348.15	$2.22 \cdot 10^{-3}$	$3.45 \cdot 10^{-3}$	$7.13 \cdot 10^{-3}$
353.15	$2.63 \cdot 10^{-3}$	$3.98 \cdot 10^{-3}$	$7.92 \cdot 10^{-3}$
358.15	$3.18 \cdot 10^{-3}$	$4.85 \cdot 10^{-3}$	$9.17 \cdot 10^{-3}$
363.15	$3.62 \cdot 10^{-3}$	$5.73 \cdot 10^{-3}$	$1.04 \cdot 10^{-2}$
368.15	$4.22 \cdot 10^{-3}$	$6.71 \cdot 10^{-3}$	$1.15 \cdot 10^{-2}$
373.15	$4.75 \cdot 10^{-3}$	$7.65 \cdot 10^{-3}$	$1.27 \cdot 10^{-2}$

* Relative standard uncertainty of the specific conductivity is 0.1; standard uncertainty of the temperature is 0.05 K.

Molar Conductivity

The molar conductivities (Λ) of the PILs were obtained from the specific conductivities σ via Eq. (S4):

$$\Lambda = \frac{\sigma M}{\rho} \qquad \text{(S4)}$$

where M is the molar mass and ρ is the density of the PIL. Results are reported in Table S8. The density values used here for the molar conductivity determination were obtained from the linear regression using the parameters reported in Table 3 of the manuscript.

Table S8. Molar conductivity of the PIL samples*

	Molar conductivity ($S.cm^2.mol^{-1}$)		
Temperature (K)	DBUH-IM14	DBUH-TFO	DBUH-TFSI
298.15	0.060		
303.15	0.086		
308.15	0.130	0.108	
313.15	0.175	0.149	0.635
318.15	0.230	0.197	0.777
323.15	0.301	0.251	0.955
328.15	0.382	0.340	1.150
333.15	0.479	0.432	1.370
338.15	0.589	0.540	1.616
343.15	0.715	0.660	1.875
348.15	0.847	0.797	2.155
353.15	1.008	0.922	2.400
358.15	1.223	1.127	2.788
363.15	1.401	1.335	3.163
368.15	1.638	1.568	3.509
373.15	1.849	1.792	3.885

*Relative standard uncertainty of the specific conductivity is 0.12; standard uncertainty of the temperature is 0.05 K.

The VFT model can also describe the molar conductivity (Eq. S5) when the density shows a slightly linear dependence with temperature [11].

$$\Lambda = \Lambda_0 \exp\left[\frac{-B}{T - T_0}\right] \qquad \text{(S5)}$$

with Λ_0 ($S.cm^2.mol^{-1}$), B (K), and T_0 (K) adjustable parameters. The best-fit VFT parameters for the molar conductivity of the studied DBUH-PILs are listed in Table S9 and the fit curves are plotted in Figure 6b of the manuscript.

Table S9. VFT fit parameters of the molar conductivity.

PIL	T range (K)	Λo ($S.cm^2.mol^{-1}$)	B (K)	T_0 (K)	R^2
DBUH-TFSI	313 - 373	182.4	719.1	185.9	0.999
DBUH-TFO	308 - 373	358.7	992.2	185.7	0.999
DBUH-IM14	298 - 373	164.1	779.6	199.5	0.999

Ionicity

The ionicity (I_W) can be estimated using the Walden approach, according to the Eq. S6 [12,13].

$$I_w = \frac{\Lambda_M}{\Lambda_M^0} = \frac{\Lambda_M}{1/\eta} = 10^{-\left(\log\frac{1}{\eta} - \log\Lambda\right)} = 10^{\Delta W} \tag{S6}$$

Table S10. Ionicity values (I_w) estimated by the Walden approach for the studied PILs.

Temperature (K)	DBUH-TFO	DBUH-TFSI	DBUH-IM14
298			0.55
308	0.99		0.53
318	0.88	0.66	0.49
328	0.82	0.61	0.46
338	0.76	0.58	0.44
348	0.70	0.54	0.40
348	0.66	0.50	0.40
368	0.63	0.48	0.38

REFERENCES

(1) Montanino, M.; Alessandrini, F.; Passerini, S.; Appetecchi, G. B. Water-Based Synthesis of Hydrophobic Ionic Liquids for High-Energy Electrochemical Devices. *Electrochim. Acta* **2013**, *96*, 124–133.

(2) De Francesco, M.; Simonetti, E.; Gorgi, G.; Appetecchi, G. About the Purification Route of Ionic Liquid Precursors. *Challenges* **2017**, *8*, 11.

(3) Henderson, W. A.; Passerini, S. Phase Behavior of Ionic Liquid-LiX Mixtures: Pyrrolidinium Cations and TFSI- Anions. *Chem. Mater.* **2004**, *16*, 2881–2885.

(4) Gómez, E.; Calvar, N.; Domínguez, Á. Thermal Behaviour of Pure Ionic Liquids. In *Ionic Liquids - Current State of the Art*; 2015; pp 200–228.

(5) Maton, C.; De Vos, N.; Stevens, C. V. Ionic Liquid Thermal Stabilities: Decomposition Mechanisms and Analysis Tools. *Chem. Soc. Rev.* **2013**, *42*, 5963–5977.

(6) Mariani, A.; Bonomo, M.; Gao, X.; Centrella, B.; Nucara, A.; Buscaino, R.; Barge, A.; Barbero, N.; Gontrani, L.; Passerini, S. The Unseen Evidence of Reduced Ionicity: The Elephant in (the) Room Temperature Ionic Liquids. *J. Mol. Liq.* **2021**, *324*.

(7) Costa, A. J. L.; Soromenho, M. R. C.; Shimizu, K.; Marrucho, I. M.; Esperança, J. M. S. S.; Lopes, J. N. C.; Rebelo, L. P. N. Density, Thermal Expansion and Viscosity of Cholinium-Derived Ionic Liquids. *ChemPhysChem* **2012**, *13*, 1902–1909.

(8) Gardas, R. L.; Freire, M. G.; Caryalho, P. J.; Marrucho, I. M.; Fonseca, I. M. A.; Ferreira, A. G. M.; Coutinho, J. A. P. High-Pressure Densities and Derived Thermodynamic Properties of Imidazolium-Based Ionic Liquids. *J. Chem. Eng. Data* **2007**, *52*, 80–88.

(9) Gomes De Azevedo, R.; Esperança, J. M. S. S.; Szydlowski, J.; Visak, Z. P.; Pires, P. F.; Guedes, H. J. R.; Rebelo, L. P. N. Thermophysical and Thermodynamic Properties of Ionic Liquids over an Extended Pressure Range: [Bmim][NTf2] and [Hmim][NTf2]. *J. Chem. Thermodyn.* **2005**, *37*, 888–899.

(10) Crespo, E. A.; Silva, L. P.; Correia, C. I. P.; Martins, M. A. R.; Gardas, R. L.; Vega, L. F.; Carvalho, P. J.; Coutinho, J. A. P. Development of a Robust Soft-SAFT Model for Protic Ionic Liquids Using New High-Pressure Density Data. *Fluid Phase Equilib.* **2021**, *539*, 113036.

(11) Rauber, D.; Philippi, F.; Zapp, J.; Kickelbick, G.; Natter, H.; Hempelmann, R. Transport Properties of Protic and Aprotic Guanidinium Ionic Liquids. *RSC Adv.* **2018**, *8*, 41639–41650.

(12) MacFarlane, D. R.; Forsyth, M.; Izgorodina, E. I.; Abbott, A. P.; Annat, G.; Fraser, K. On the Concept of Ionicity in Ionic Liquids. *Phys. Chem. Chem. Phys.* **2009**, *11*, 4962–4967.

(13) Philippi, F.; Rauber, D.; Zapp, J.; Hempelmann, R. Transport Properties and Ionicity of Phosphonium Ionic Liquids. *Phys. Chem. Chem. Phys.* **2017**, *19*, 23015–23023.

(14) Miran, M. S.; Kinoshita, H.; Yasuda, T.; Susan, M. A. B. H.; Watanabe, M. Physicochemical Properties Determined by ΔpKa for Protic Ionic Liquids Based on an Organic Super-Strong Base with Various Brønsted Acids. *Phys. Chem. Chem. Phys.* **2012**, *14*, 5178–5186.
